# Gut Flora-Mediated Metabolic Health, the Risk Produced by Dietary Exposure to Acetamiprid and Tebuconazole

**DOI:** 10.3390/foods10040835

**Published:** 2021-04-12

**Authors:** Jingkun Liu, Fangfang Zhao, Yanyang Xu, Jing Qiu, Yongzhong Qian

**Affiliations:** 1 Institute of Quality Standards & Testing Technology for Agro-Products, Key Laboratory of Agro-Product Quality and Safety, Chinese Academy of Agricultural Sciences, Key Laboratory of Agri-Food Quality and Safety, Ministry of Agriculture and Rural Affairs, Beijing 100081, China; liujk918@163.com (J.L.); xuyanyang@caas.cn (Y.X.); qianyongzhong@caas.cn (Y.Q.); 2 Analysis & Testing Center, Chinese Academy of Tropical Agricultural Sciences, Haikou 571101, China; zhaofangfangfang@163.com

**Keywords:** gut flora, pesticide residues exposure, metabolic syndrome, health risk, metabolite

## Abstract

The low-level and long-term exposure of pesticides was found to induce metabolic syndrome to mice. Metabolic pathways and mechanisms were investigated by detecting gut flora with metabolites, host circulation, and their interrelations. Results showed that the abundances of flora species and their metabolism were altered, consequently leading to metabolic disorders. A correlation analysis between gut flora and their metabolic profiling further explained these changes and associations. The metabolic profiling of host circulation was also performed to characterize metabolic disorders. The associations of host circulation with gut flora were established via their significantly different metabolites. Alterations to the liver metabolism clarified potential pathways and mechanisms for the disorders. Metabolic disorders were evidently released by dietary and micro-ecological intervention, directly proving that gut flora comprise a vital medium in metabolic health risk caused by pesticide exposure. This work supplied theoretical bases and intervention approaches to body metabolic problems caused by pesticide exposure mediated by gut flora.

## 1. Introduction

Gut flora are profoundly associated with human health, and they function as inducers and even driving forces in diseases, such as metabolic disease, mental illness, degenerative diseases, and even cancers [1]. Thus, researchers now view the gut microbiome as a new metabolic organ and are paying increased attention to it. Among those aforementioned diseases, metabolic dysfunction includes a board spectrum of diseases such as nonalcoholic fatty liver disease (NAFLD), type 2 diabetes (T2D), obesity, and atherosclerosis [2]. These diseases and their related complications are some of the leading causes of the low quality of life and even death of humans.

Besides poor eating habits and mental stress, dietary contamination promotes gut flora alteration and usually poses a direct hazard to gut flora [3]. It is also an essential driving force of dysfunction in gut flora. Pesticide residue is an inevitable problem in dietary contamination because pesticides are still the most effective countermeasures to ensure food supply and security. Pesticide residues in food are normally in trace amounts level; thus, they do not exert acute toxicity to the body. The long-term intake of pesticides with a diet potentially threatens human health [4], although residue levels are normally not above the limit. Evidence has supported that pesticides promote chronic or metabolic health problems, and they should not be an underestimated public health issue [5].

Pesticides have been reported to affect body health and metabolism. For example, pesticides were found to affect bile acid metabolism and to lead to body inflammation [6]. Exposure to pesticide residue was also found to alter the composition of gut flora, thus triggering host metabolism changes and even diseases [7]. Though newly developed and widely used pesticides usually have a low toxicity, the influence of the long-term intake of low levels of pesticide residues on health should not be underestimated. Moreover, combined residues usually exist in foods due to the mixed use of various pesticides in agriculture. The effects of combined pollution on gut flora are ambiguous due to different bioactivities. Therefore, combined pollution may bring increased uncertainty in gut flora, thus deserving further study to understand the relationship among pesticides, gut flora, and diseases.

Gut flora have a large diversity, and their metabolism alteration becomes more intricate when influenced by pesticides. Understanding these changes and developing associated analyses are the basic and effective ways to understand the relationship among contamination, gut flora, and disease. Here, mice were exposed to a combination of pollution, a low-level residue of a type of insecticide, and a type of fungicide. Mice physiological changes were first observed, and then alterations in gut flora and their metabolism were investigated. The results closely linked the alterations between the gut flora and physiological changes. Gut flora and their metabolism were thoroughly explored to understand the relationships and mechanism among pesticides, host physiological changes, and intestinal microflora. Furthermore, the host’s circulating metabolism was analyzed, and its association with gut flora was determined. Alterations in the metabolism pathways in the host liver were also examined. Finally, interventions to the gut flora proved that dietary pesticide exposure mediated the risk of on host metabolic health.

## 2. Materials and Methods

### 2.1. Animal Experiment

Three-week-old male Kunming mice were purchased from Vital River Laboratory Animal Technology Co., Ltd. (Beijing, China). All of the used chemical reagents were purchased from Guoyao Co., Ltd. (Shanghai, China).The experiment was carried out in accordance with the Animal Welfare Committee guidelines for laboratory animals. After one week of acclimation, the mice were randomly divided into five groups and subjected to treatments of W (tebuconazole in corn oil), D (acetamiprid in corn oil), DW (tebuconazole and acetamiprid in corn oil), control check (CK—only corn oil), and BL (only water). Following the literature [8], mice of W, D, and DW treated groups were, respectively, exposed at the levels of 1.35, 3.15, and (1.35 + 3.15) mg/(kg bodyweight) per day for 13 weeks, with 25 μL of solution for each mouse. The mice were maintained under a 12:12 h light/dark cycle at 25–28 °C and allowed free access to feed and water. The padding in the cages was regularly replaced per week to keep the humidity at 40–70%.

### 2.2. Sampling

After 12 weeks of treatment, the mice were transferred into metabolic cages at the same time of day. Their urine and feces were collected, immediately flash-frozen in liquid nitrogen, and stored at −80 °C until processing. After 13 weeks of treatment, four DW groups (32 mice) and one CK group (eight mice) mice were fed continuously, whereas the others were euthanized by CO_2_ asphyxiation and cervical dislocation. Blood was collected and centrifuged to obtain serum. Samples for the hematoxylin and eosin staining of the colon and liver were collected and fixed in 10% formalin. The rest of the liver was cut into several pieces, immediately flash-frozen in liquid nitrogen, and stored at −80 °C.

### 2.3. Measurement of Mouse Glucose Tolerance

A blood glucose meter (Accu-Chek, Roche Diagnostics Inc., Basel, Switzerland) was used to measure the tail-vein blood glucose levels. At approximately 13 weeks of exposure, the glucose tolerance (GT) of the mice was examined. After the mice were fasted for 16 h (water was available), they received a gavage of glucose (2 g/kg bodyweight). Blood glucose was measured at 0, 30, 60, and 120 min after the glucose loading.

### 2.4. Measurement of Mouse Insulin Resistance

A homeostasis model assessment was employed to calculate and estimate the insulin resistance (IR) of the mice. At the end of the exposure experiment, the mice were fasted overnight and the fasting blood glucose (FBG) was measured using the blood glucose meter. Then, fasting serum insulin (FINS) was determined using an ELISA kit (Mercodia) after the serum was obtained. Finally, the IR index was calculated in accordance with HOMA–IR (homeostasis model assessment–IR) = FBG (mmol/L) × FINS (mIU)/22.5.

### 2.5. Serum Biochemical Assays

Serum biochemical parameters, alanine aminotransferase (ALT), aspartate aminotransferase (AST), total protein (TP), and albumin (ALB) were assessed using a biochemical analyzer (Hitachi 7100, Tokyo, Japan).

### 2.6. Inflammatory Factor Assays

Inflammatory factors were investigated to evaluate the inflammatory status of the mice. The lipopolysaccharide (LPS), macrophageinflammatoryprotein-1 (MCP-1), interleukin-1β (IL-1β), IL-6, IL-10, and tumor necrosis factor-α (TNF-α) levels in serum or feces were assayed using a commercial ELISA kit in accordance with the product’s instruction manual.

### 2.7. Evaluation of In Vivo Intestinal Permeability

For the observation of intestinal permeability, blood was obtained from the overnight-fasted mice (0 h). Then, the mice were administered with 600 mg/kg of FITC (Fluorescein Isothiocyanate) -dextran (MW 4000, Sigma-Aldrich) by gavage. Two hours later, the blood and urine were collected and detected via fluorometry, with a 0-h sample as background (Excitation wavelength: 490 nm; Emission wavelength: 520 nm).

### 2.8. Histological Observation

The fixed liver and colon were paraffin-embedded, dewaxed, rehydrated, and then stained with HE (hematoxylin eosin) for histopathological analysis. The staining was visualized with an ECHO^®^ microscope (RVL-100), and the observations were approved by a histopathologist.

### 2.9. Targeted and Untargeted Metabolic Profiling Analysis

The alterations that occurred to the mouse physiology after exposure were investigated using metabolic techniques on the basis of high-resolution mass spectroscopy (QE plus, Thermo Fisher, Waltham, MA, USA), and the samples involved the serum, feces, and liver. The involved methods are described in Supplementary File S1 (Methods for Metabolic Profiling Analysis).

### 2.10. Gut Flora Analysis

The gut flora in the frozen feces were analyzed using the 16S rRNA gene sequencing method in accordance with the procedures of Novogene Bio-Information Technology Company (Beijing, China; Supplementary File S1: Sequencing method).

### 2.11. Intervention to Metabolic Disorders

After approximately 13 weeks of exposure, the four DW groups and one CK group were fed continuously for 4 weeks. The four DW groups were treated with fecal microbiota transplantation (FMT), fructooligosaccharide (FOS), FMT and FOS, and only water. Detailed procedures and methods are shown in Supplementary File S1.

### 2.12. Correlation and Statistical Analysis

Correlation analysis among datasets was conducted based on the Spearman coefficient, and the results are exhibited in a network map via Cytoscape (3.8.0). Differences between data groups were investigated with a one-way ANOVA in SPSS (19.0). Diversity analysis was carried out on the OmicShare data processing platform (https://www.omicshare.com, accessed on 1 March 2021, Guangzhou, China) and with the R language.

## 3. Results

### 3.1. Effects of Exposure on Mouse Bodyweight

The results in Figure S1 show that the weight gradually increased over the course of exposure. Approximately three weeks later, the bodyweight difference extensively increased. Compared with the control check group, the acetamiprid, tebuconazole, and combination groups showed a 19.35–22.06% increase in bodyweight on average. This finding indicated that obvious obesity trends or even obesity occurred in the exposure-treated mice. Meanwhile, the bodyweight of the blank group showed no significant difference with that of the CK group, indicating that the corn oil used as a reagent in the administration hardly affected the basal metabolism of the mice and that the bodyweight difference was mainly caused by the pesticides. Bodyweight is only the phenotype of obesity, and metabolic abnormalities occur in physiological reactions [9]. Physiological observation and metabolic investigation could be performed in further studies.

### 3.2. Effects of Exposure on Glucose Tolerance and Insulin Resistance

**Figure 1:**
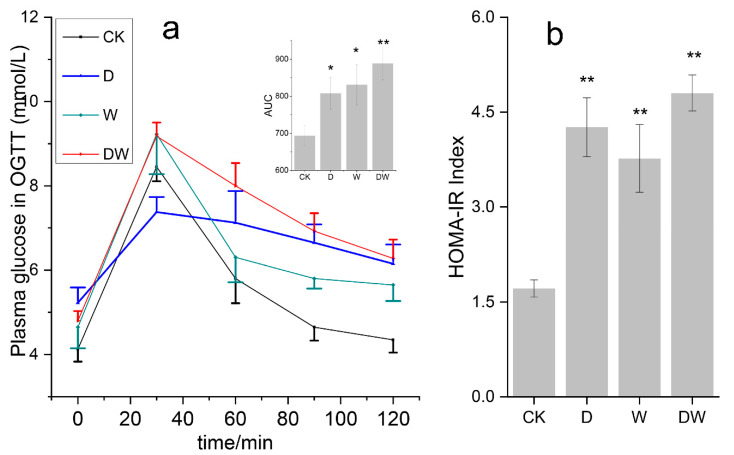
Effects of pesticide exposure on mice glucose tolerance (**a**) and IR (insulin resistance) degree (**b**); a high value of homeostasis model assessment–IR (HOMA–IR) means high degree of IR) (*n* = 4). Data are expressed as the mean ± SEM (* *p* < 0.05; ** *p* < 0.01). CK: control check group; D: acetamiprid treated group; W: tebuconazole treated group; DW: combination treatment group.

Long-term obesity usually occurs along with IR, and it is the link of many metabolic diseases [10]. The results revealed that the FBG in the treated groups slightly increased, though to less than 6 mmol/L (Figure 1a). The mouse plasma glucose rapidly increased after glucose intake in all the groups. Then, the glucose concentrations slowly decreased in the following 90 min. The treated groups maintained a higher plasma glucose than the CK group at the end of 120 min. This finding suggested that the plasma glucose in the treated mice spent more time restoring to normal, indicating that pesticide exposure lowered the capacity of the body to regulate glucose [11].

A high IR meant that the target cells lowered the sensitivity to insulin. The test results (Figure 1b) suggested that the IR indices in the treated groups were nearly one-to-three times more than those in the CK group. Significant differences in IR index levels were observed in mice pressured using two pesticides. The results of the present study revealed that dietary pesticide exposure induced IR in the mice. The effect of combined pesticides on IR was significantly higher than that of their single components, indicating that the combination exposure enhanced the health risk compared with single component. This finding revealed that combination exposure strengthened the exposure effects compared to single components.

### 3.3. Serum Biochemistry Analysis

IR and the disorder of carbohydrate metabolism occurred, indicating that many aspects of metabolism were affected. Pesticide exposure obviously led to a significant increase in serum cholesterol (Figure S2) and a decrease in high-density lipoprotein cholesterol (HDL-C) in all the treated groups. Similar increasing trends were also obtained in serum triglyceride (TG). The combined exposure group displayed significantly higher serum TG levels than any single exposure group. Therefore, the metabolic abnormalities caused by pesticide exposure could induce a risk of cardiovascular disease and fatty liver to the body.

In this work, liver function was investigated and evaluated by using related indices (Figure S3) such as aminotransferase (ALT, AST, and ALP) and serum protein (TP and ALB) [12]. Aminotransferases, especially ALT and AST, were enhanced at different degrees by pesticide exposure, indicating that minor injuries occurred in the liver. The concentration changes in serum protein were slighter in the exposure than those in aminotransferase. The evaluation results revealed that liver was slightly impaired and mild dysfunction may have occurred in the exposure [13]. Moreover, these abnormalities were all found in different exposure groups, while no obvious joint effects were obtained in the combination exposure group.

### 3.4. Evaluation of Mouse Inflammation

**Figure 2:**
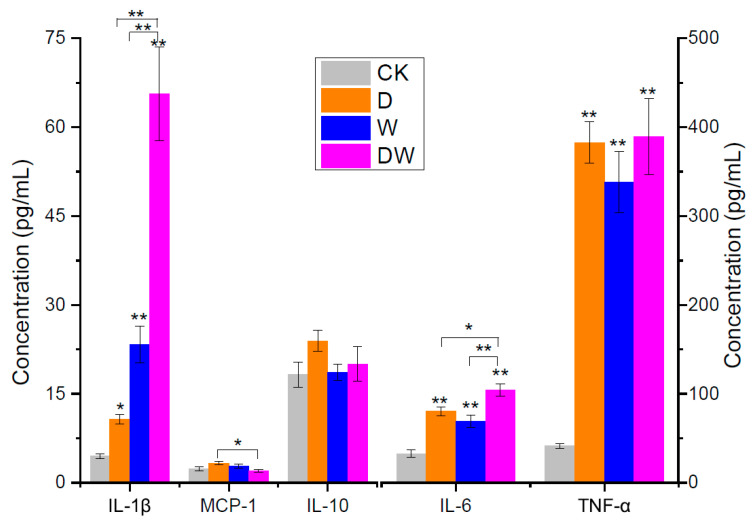
Concentrations of mice serum inflammation factors (*n* = 4). Data are expressed as the mean ± SEM (* *p* < 0.05; ** *p* < 0.01).

According to previous report [14], chronic and systemic inflammatory responses trigger the development of IR. Thus, the inflammation status of the mice is discussed in this section. The levels of several important inflammatory mediators in serum are exhibited in Figure 2. TNF-α is one of the most crucial factors in IR formation; it works by inhibiting insulin signal transduction. A previous study reported that the antibody against TNF-α evidently improved insulin sensitivity and alleviated IR [15]. Single and combination exposures led to a massive increase in TNF-α by almost 10-fold. For example, exposure to tebuconazole increased TNF-α from 35.7 EU/L (CK) to 338.10 EU/L. No significant joint action effect was obtained in the combination exposure group compared with the single-component group.

Interleukin is a large class of cell factors that exerts considerable effects to the body’s inflammatory reaction and immunity. Numerous studies have shown that IL-1, IL-6, IL-8, and IL-10 exerted substantial effects on IR. Increased levels of IL-1β and IL-6 were detected in patients with T2D and obesity, and they promoted the inflammatory process. MCP-1 is another important promoter that acts as a kind of inflammatory chemokine and contributes to IR. Though MCP-1 did not intensively respond, a significant increase of its concentration was observed. Different from these inflammatory mediators, IL-10 is a well-recognized multifunctional factor, and it was also negatively-related with IR in this work. No evident concentration differences were observed between CK and exposure groups. Overall, the observation of these cell factors indicated that dietary exposure to pesticides induced inflammation, and it is an important basis of IR.

### 3.5. Detection of Lipopolysaccharide and Intestinal Permeability

LPS is usually the reason of inflammation and mainly produced by intestinal flora [16]. The results (Figure S4b) show that increased FITC-dextran amount was observed in all the treated groups, indicating that the intestinal permeability significantly increased, especially in the tebuconazole and combined groups. Tebuconazole exhibited a more obvious effect than acetamiprid, and no significant difference was found between the tebuconazole and combined groups. Serum LPS increased in exposure groups, and a facilitation effect was obtained in the combined exposure group (Figure S4a). The coordination effects of combination exposure were exhibited in the results of investigation on LPS and intestinal permeability; significant differences occurred between combined and single actions. Pesticide exposure increased the intestinal permeability, and the resulting enhanced transfer of harmful gut flora and metabolites to the blood led to increased physiological dysfunctions, such as low-grade inflammation.

### 3.6. Tissue Histology

**Figure 3:**
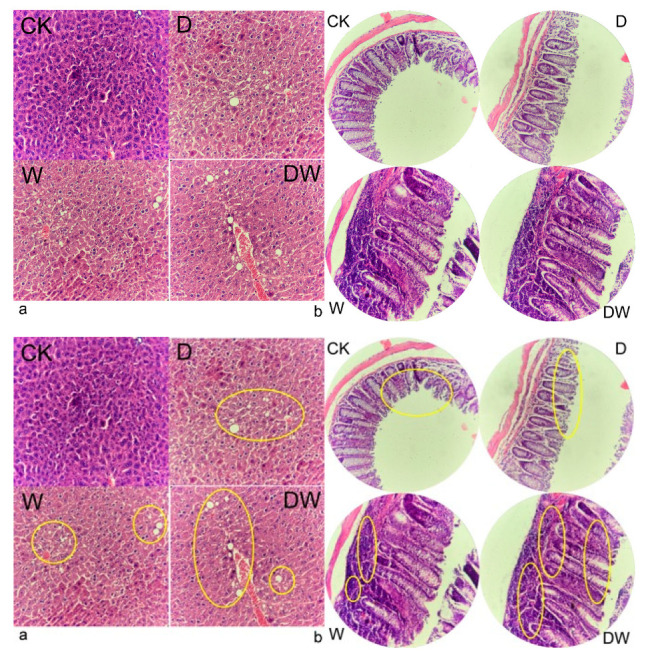
HE (hematoxylin eosin) staining of mice liver sections (**a**) and colon sections (**b**).

Host tissue histology showed that lipid droplets were gradually formed and NAFLD was presented in the treated groups (Figure 3). The HE staining of colon slices showed that aggravated inflammatory cell infiltrates occurred in the treated groups, especially in the tebuconazole and combined exposure groups. The cell structure of intestinal villi was loosened and intestinal barrier function was impaired in acetamiprid group, which enhanced intestinal permeability. The results proved that dietary pesticide exposure leads to NAFLD and leaky gut. The metabolism health risks of chronic dietary pesticide exposure were confirmed.

### 3.7. Effects of Pesticide Exposure on the Diversity of Gut Flora Community Structure

As mentioned above, investigating the changes in the intestinal flora caused by pesticide exposure was necessary in this work [17]. The diversity of gut flora was investigated at the alpha and beta levels. The results of the alpha diversity investigation (Shannon index) showed that no significant difference was found between the CK and treated groups, indicating that the species diversity of gut flora within a community were not destroyed by pesticide exposure (Figure S5a). Nonmetric multidimensional scaling analysis was decided to be an appropriate method for calculating the beta diversity of the gut flora community. Figure S5b illustrates that relatively different beta diversities occurred in different groups of treated mice, thus revealing that the gut flora of mice had a unique response to the exposure of different pesticides and that their communities were differently altered. The stress value of the model was 0.093, revealing that the model could accurately stimulate the actual samples.

### 3.8. Species Changes in Gut Flora

**Figure 4:**
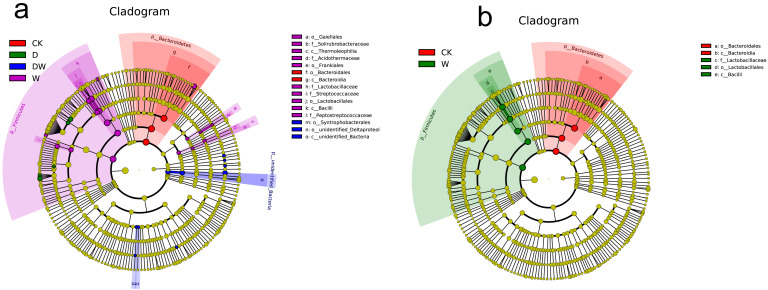
Evolutionary branches in LDA (Linear Discriminant Analysis) effect size (LEfSe) analysis to mice gut flora: (**a**) LDA score is 3 and (**b**) LDA score is 4, *n* = 4).

Similar to the reports (Figure S6a), Bacteroidetes and Firmicutes constituted more than 95% of the gut flora. There were approximately 3–4-fold more Bacteroidetes/Firmicutes in the CK group than in the treated groups, thus promoting host obesity [18]. The results in Figure 4 and Figure S5c,d show that most significantly different gut flora species (around 18 categories) were obtained in the tebuconazole group according to the LDA (Linear Discriminant Analysis) effect size (LEfSe) analysis when the LDA threshold was 3. In addition, the families of Peptostreptococcaceae and Lactobacillaceae showed abundant microbes in the gut, and these microbes were accumulated in the tebuconazole group. They were greatly affected in the combined and acetamiprid groups. Significantly different flora were only found between the tebuconazole and CK groups when the LDA threshold was 4, indicating that the gut flora were mostly affected by tebuconazole in all exposure groups. Compared with single components, the combination exposure did not show evident promotion effects. Further investigation was focused on the quantity alteration in the flora and their distribution in the levels of family or genus.

The gut flora species of different groups at the genus level were investigated (Figure S7). Compared with the CK group, acetamiprid enhanced the abundance of many non-dominant species, such as *Ruminiclostridium*, *Roseburia*, *Lachnoclostridium*, and *Marvinbryantia*. The dominant species, *Alistipes*, *Blautia*, unidentified *Ruminococcaceae*, and *Oscillibacter* were also mildly increased. These species contain both harmful and benefit microflora. Compared with the CK group, *Lactobacillus*, *Klebsiella*, *Streptococcus*, *Romboutsia*, *Mitsuokella*, *Enterococcus*, and *Sphingomonas* were upgraded by tebuconazole, while *Alloprevotella*, *Bacteroides*, and *Muribaculum* were decreased. The intestinal microecology may have been deteriorated due to the accumulation of major pathogenic bacteria. For example, the accumulation of *Enterococcus* leads to gut inflammation and lowers the butyrate-maker flora and butyrate in the gut [19]. *Klebsiella* and *Streptococcus* are also typical infectious bacteria [20], while *Alloprevotella*, *Bacteroides*, and *Muribaculum* are anti-inflammatory bacteria that produce short-chain fatty acids (SCFAs). A decrease in these bacteria lowers the anti-inflammatory and immune capacity of the host [21]. Thus, dietary tebuconazole exposure exerted more negative effects on the gut flora, thus disrupting the healthy gut flora of the host and posing a metabolic risk to them. The combination exposure of pesticides exerted multiple effects on the gut flora. Harmful bacteria, such as *Helicobacter* and *Lachnospira*, and beneficial bacteria, such as *Parabacteroides* and *Akkermansia*, were all enhanced. Meanwhile, absolutely dominant bacteria, including *Alloprevotella* and *Bacteroides*, were all decreased by the exposure. Therefore, pesticide exposure’s influences on the gut flora were multifaceted, and disorders were obtained after the disruption to the absolutely dominant species of bacteria. Overall, tebuconazole disrupted the gut flora more than the other treatments, and no significant synergy effects were obtained from the combination exposure group. This finding was consistent with the result of the LEfSe analysis. LPS produced by Enterobacteriaceae and *Desulfovibrionaceae* was reported to be approximately 1000-fold higher than that from other bacteria [22], and it was remarkable in causing chronic inflammation. The abundances of *Desulfovibrionaceae* in the acetamiprid, tebuconazole, and combined groups were approximately 3.5, 1.5, and 10 times, respectively, that in the CK group, and pesticide treatments significantly enhanced these abundances in the tebuconazole and acetamiprid groups. The abundances of Enterobacteriaceae in these groups were 5.3, 64, and 3.2 times, respectively, higher than that in the CK group (Figure S6b). They were highly accumulated by pesticide exposure, especially in the tebuconazole and combined groups, which could extremely promote endotoxin accumulation in the gut.

### 3.9. Metabolic Profiling of Gut Flora

Depending on the gut structure [23], the metabolites of the gut flora are the main media of interaction between the flora and the host through blood absorption [24]. Thus, identifying the altered metabolites of the gut flora comprise an essential means to investigate the effects of pesticide exposure on host physiological activity. Metabolic analysis was decided to be an effective method to obtain the changes in gut flora metabolism between the CK and exposure groups.

On the basis of the metabolites, the samples of different exposure groups could only be significantly distinguished by an unsupervised learning algorithm—principal component analysis (PCA; Figure S8). The results indicated that circulating metabolism changes and differences occurred when hosts were stressed by different pesticides. Furthermore, differential metabolites were yielded via OPLS-DA (Orthogonal Partial Least Squares Discrimination Analysis) model under the following statistical conditions: VIP > 1, FC > 2 (or <0.5), FDR < 0.05, and *p* < 0.05. Compared with CK, 37, 43, and 32 different metabolites (Table S1) respectively in the acetamiprid, tebuconazole, and combination groups were investigated and used for further investigation. Different metabolites were also obtained in the single and combined exposure groups to evaluate the gut flora response to pesticide exposure.

In this work, pesticide exposure involved many significant metabolite variations in the gut. Trimethylamine N-oxide (TMAO) comes from the metabolism of intestinal microflora, and it is highly related to cardiovascular diseases. It is the main risk factor of atherosclerosis [24]. In the present study, the acetamiprid and combined groups showed considerable increases in TMAO in the gut by approximately thousands of times more than the CK group. However, this metabolite between the CK and tebuconazole groups did not significantly differ. Thus, acetamiprid was evidently an important dietary contaminant, and its exposure led to the risk of cardiovascular diseases via the adjustment of the gut flora metabolism. Spermidine, the most effective polyamine in preventing lipid peroxidation, was lowered in the acetamiprid group. On the contrary, putrescine, another type of polyamine, increased by approximately 100 times when the mice were exposed to acetamiprid, thus considerably enhancing the risk of leaky gut and colitis. These significant variations in polyamines did not occur in the tebuconazole and combined groups. Imidazole propionate and imidazoleacetic acid were the metabolites of the gut flora. A significant decrease in imidazoleacetic acid was yielded in all pesticide-exposed groups. They were homologous in structure, with only a difference in CH_2_. Imidazole propionate and imidazoleacetic acid were all gut flora metabolites of histidine from totally different metabolic pathways. Imidazole propionate occurred with the action of histidine ammonia lyase, while imidazoleacetic acid was the final metabolite of histamine, which was obtained from histidine treated with histidine decarboxylase. Imidazole propionate was reported to cause T2D by disrupting GT and insulin signaling [25]. No physiological function of imidazoleacetic acid was ever reported, but speculations could be made on the basis of their extremely similar molecular structure. Extensive work is necessary to identify how gut flora work in the pathway of imidazoleacetic acid production and what determines the metabolic method of histidine.

The metabolism of tryptophan involves a number of compounds, especially indole derivatives. Many of them, including 3-indoxyl sulphate, 3-indolelactic acid, 5- hydroxyindoleacetic acid, and indole-3-acrylic acid, were the significantly different metabolites distributed in the content sequence of control > acetamiprid > combination > tebuconazole. These metabolites played substantially important signaling roles in regulating host physiological activities, such as enhancing mucosal homeostasis by alleviating intestinal permeability (possibly mediated by the pregnane X receptor), suppressing appetite, secreting insulin, and slowing gastric emptying by inducing the release of glucagon-like peptide 1 in enteroendocrine L-cells [26]. A decrease in these metabolites by pesticide exposure could elevate the health risk to the host. The results suggested that tebuconazole lowered indole derivatives more than acetamiprid. Many other gut intestinal metabolites that have been proven to act on host metabolic activity were affected by pesticide exposure. For instance, 12, 13-DiHOME is a kind of gut lipid that activates the brown adipose tissue of the host, regulates fat metabolism, and lowers the host risk of heart disease and diabetes as a metabolic signal [27]. In the present work, only single-component exposure significantly decreased 12, 13-DiHOME in the host gut.

### 3.10. Association of Gut Flora with Their Metabolism

**Figure 5:**
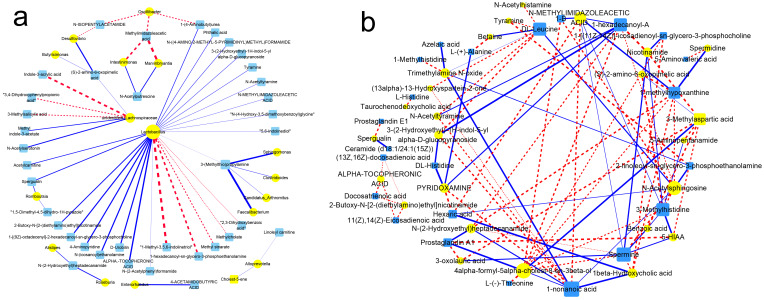
Associations of gut microbial species with their metabolites (**a**) and associations of gut microbial metabolites with host circulation metabolites (**b**). Yellow round nodes: different genera of the intestinal microbiota; blue square nodes: metabolites of the microbiota; red dashed lines: negative correlation; blue solid lines: positive correlation. The width of lines indicates the magnitude of correlations, from −0.7 to −1.0 or from 0.7 to 1.0 (Spearman). The size of nodes represents how many correspondences of this element were involved with another type of element.

Under the pressure of pesticides, the gut flora communities were affected, and the metabolites were altered correspondingly. The relevance between altered gut flora and their metabolites were analyzed, and the results are shown in Figure 5a. A high relationship (r > 0.7 or r < −0.7 and *p* < 0.05) between the flora at the genus level and their metabolites was subjected to network analysis, as seen in Figure 5a.

The genera of *Lactobacillus*, *Alloprevotella*, *Alistipes*, *Roseburia*, *Enterorhabdus*, *Romboutsia*, *Faecalibacterium*, and *Clostridioides* were involved in high relevance, and *Lactobacillus* was associated with most of the metabolites, including being positively related with N-(2-acetylphenyl)formamide and 1,5-fimethyl-4,5-dihydro-1H-pyrazole and being negatively related with 1-methyl-3,5,6-indolinetriol and 1-hexadecanoyl-sn-glycero-3-phosphoethanolamine. Methylimidazoleacetic acid was decreased by *Intestinimonas* and *Marvinbryantia*, while N-acetylputrescine was totally different with it. In addition to methylimidazoleacetic acid, N-isopentylacetamide and 1-(4-Aminobutyl)-urea were also negatively related with *Oscillibacter*. Though *Butyricimonas* and *Desulfovibrio* exerted different effects on host, they were all positively related with (S)-2-amino-6-oxopimelic acid. *Desulfovibrio* still related with the host more closely than *Butyricimonas*. Gut flora and metabolites were all groups with large amounts and complexity, and their correlations were also extremely intricate. The pesticides altered the intestinal microflora, which changed the metabolism of the flora. This phenomenon mediated the effects of pesticides on the host’s physiological activity. Exploring their relationships is an effective way to investigate the effects of gut flora on a host’s metabolic syndrome [28,29].

### 3.11. Effects of Exposure on Host Circulation Metabolism

Groups were distinguished in accordance with the identified metabolites in serum, as shown in Figure S9. Wide varieties of significantly different metabolites were identified, mainly including amino acids and their derivatives, free fatty acids and their methyl esters, phospholipids, nucleotides, carbohydrates, hormones, and other physiological metabolic compounds (Figure S10 and Table S2). As shown in Table S3, different alterations in amino acids were observed; for example, the contents of branched-chain (valine and leucine) and aromatic amino acids (phenylalanine) in the serum showed variable degrees of elevation in all the treated groups. Threonine was increased by pesticide exposure, and no obvious change trend was found in the amino acid derivatives. As reported, the disorders of fat and carbohydrate metabolism also exhibited dysfunctions of body physiological activities, especially in chronic metabolism diseases, such as obesity, T2D, and NAFLD. Fatty acids and methyl esters were accumulated by around 2–11-folds in the exposure groups. Interestingly, all of them were unsaturated fatty acids, such as docosadienoic acid, docosatetraenoic acid, docosapentaenoic acid, and docosatrienoic acid. (5R)-5-((1S)-1,2-dihydroxyethyl)-alpha-D-lyxopyranose,5-O-alpha-L-arabinofuranosyl-alpha-L-arabinofuranose and methyl 6-deoxy-2,3-O-isopropylidene-alpha-L-mannopyranoside were significantly increased by pesticide exposure in the treated groups by up to approximately 3.3–5 times. Phosphatidylcholine decreased in the pesticide-exposed groups, whereas lyso PC (phosphatidylcholine) increased in the treated groups. These were the important phospholipid compounds in the serum. The results obtained in the present work were not totally consistent with those from previous reports, as different results about these phospholipid compounds in a study on metabolic disorders have been reported. Phospholipid-derived compounds undoubtedly play considerably important roles in physiological activities. For example, PC decreases blood fat and peroxide and demonstrates positive effects on the liver and heart, while LPC is an important pre-inflammatory factor of arteriosclerosis [30]. Spermine showed a 0.38–0.73-fold decrease in the present study and lowered the risk of T2D [31]. 9-methyluric acid and uric acid were differently affected by acetamiprid and tebuconazole, and they were also reported to be closely related with obesity and T2D [32]. 7-alpha-hydroxy-17alpha-methyltestosterone was the detected androgen in the serum, and it decreased in the acetamiprid and combined groups but not in the tebuconazole group, indicating that dietary acetamiprid exposure also affected the reproductive endocrine system.

### 3.12. Association between Metabolites of Gut Flora and Host Circulation

A co-inertia analysis (CIA) was performed to find a covariation between serum metabolites and gut microbiota metabolites, as well as further investigate whether the altered abundance of host metabolites correlated with the altered gut flora (Figure S11 and Table S4) [28]. Figure S11 demonstrates that the representativeness of the CIA model is obviously reflected by the first two axes, which exhibit most of the shared features of the metabolites of gut flora and serum. A high consistency was obtained between the two datasets of the metabolites of the flora and serum. The relevance of the two metabolism datasets was also significant. A correlation analysis between the metabolites of gut flora and host serum exhibited quantitative relationships of the significant compounds (Figure 5b). The trimethylamine N-oxide, N-acetylsphingosine, and betaine in the gut enhanced the leucine in the serum, while 3-(2-hydroxyethyl)-1H-indol-5-yl alpha-D-glucopyranoside, N-acetyltyramine, (13alpha)-13-hydroxyspartein-2-one, N-acetylhistamine, tyramine, and 5,6-indolinediol lowered it. Unsaturated fatty acids, except for arachidonic acid, were mostly inhibited by 2-butoxy-N-(2-(diethylamino)ethyl)nicotinamide and alpha-tocopheronic acid. Prostaglandin comes from the metabolism of arachidonic acid, and its abundance in the serum was significantly enhanced. The results of the observation proved that in the treated groups, more arachidonic acid was metabolized into prostaglandin, and its content decreased. N-methylimidazoleacetic acid, N-acetyltyramine, pyridoxamine, 3-(2-hydroxyethyl)-1H-indol-5-yl alpha-D-glucopyranoside, and nicotinamide decreased in the gut of the treated mice, whereas the contents of 4alpha-formyl-5alpha-cholest-8-en-3beta-ol, N-(2-hydroxyethyl)heptadecanamide, and N-acetylsphingosine increased. The above two groups of compounds positively and negatively affected the PCs in the host serum, respectively. Ceramide, spermine, and other amino acids related to the dysfunction of host metabolites all showed a strong correlation with the significantly different compounds in the gut. Correspondences between host and gut flora were established based on the metabolites, and they became clearer and closer as more action pathways or new metabolites were identified.

### 3.13. Host Liver Metabolism and Gut–Liver Dialogue under Pesticide Exposure

**Figure 6:**
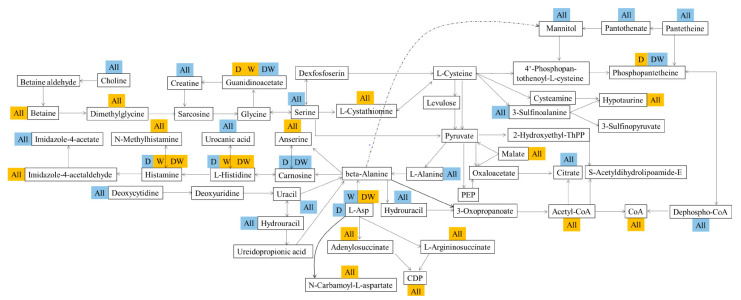
Enriched metabolic pathways in mice liver according to the announced metabolites. orange: upregulation; blue: downregulation; ‘all’ represented as D, W, and DW.

The significantly different compounds obtained in the liver metabolism research were subjected to the KEGG (Kyoto Encyclopedia of Genes and Genomes) metabolic database to map and analyze the involved pathways, and the results are exhibited in Figure 6 and Figure S12. PCA showed that pesticides exerted significantly different effects on the body metabolism (Figure S13). The affected pathways mainly involved amino acid and derivative metabolism, glycerophospholipid and fatty acid metabolism, and vitamin and nucleotide metabolism. Purine metabolism, glycerophospholipid metabolism, and vitamin B6 metabolism were shared by three different treated groups. Moreover, many pathways were only intervened by the joint exposure group. For example, beta-alanine metabolism and glycine, serine, and threonine metabolism were intensively affected by the combined stress of tebuconazole and acetamiprid.

The intervened pathways were integrated together in accordance with the shared metabolites. In the metabolism of cholic acid, a decrease in choline was observed in the three treated groups, and choline deficiency impaired PC synthesis, very-low-density lipoprotein synthesis, and hepatic lipid export [33]. Thus, this disorder posed a NAFLD risk to the host. The results were consistent with the detection of serum components. Meanwhile, increased betaine was detected, and it can be speculated that the activity of choline oxidase was enhanced because betaine was obtained from the oxidation of this enzyme. However, the reasons for the enhancements of this enzyme were unclear. A high content of betaine led to the condition for TMAO formulation, and the results were proven in previous observations. Histidine could be converted into urocanic acid or histamine via two different pathways, and an abnormality in histidine and urocanic acid was found when the host was stressed by tebuconazole. The results indicated that histidine metabolism was intensively intervened. When the body was stressed by tebuconazole, the downregulation of urocanic acid, the upregulation of histidine, and the accumulation of histamine occurred. Histamine was further reacted into N-methylhistamine and imidazoleacetic acid through different enzymes. In this work, more N-methylhistamine and less imidazoleacetic acid were the results of the alteration. More histamine was obviously oxidated into N-methylhistamine. Histamine is a type of key conductive chemical and one of the most widely studied inflammatory mediators that lead to inflammation and allergies to tissues. N-methylhistamine is the major metabolite of histamine produced in mast cells. Increased N-methylhistamine levels are typically associated with anaphylaxis and mastocytosis [34]. Upgraded N-methylhistamine was detected in the blood in the above process. Thus, the histamine and N-methylhistamine in the liver could be the significant reasons behind mastocytosis and hepatitis. The downstream nucleotide metabolites of L-Asp, L-argininosuccinate, adenylosuccinate, and CDP (Cytidine diphosphate) were upregulated in all the treated groups. On the contrary, the upstream nucleotide metabolites of beta-alanine were all downregulated. Many other metabolites were also annotated into different pathways. However, an insufficient effective abnormality was found when formulating clear pathways. This finding may be resolved by detecting and identifying more metabolites in the analysis.

### 3.14. Effects of Interventions on Physiology of Pesticide-Exposed Mice

**Figure 7:**
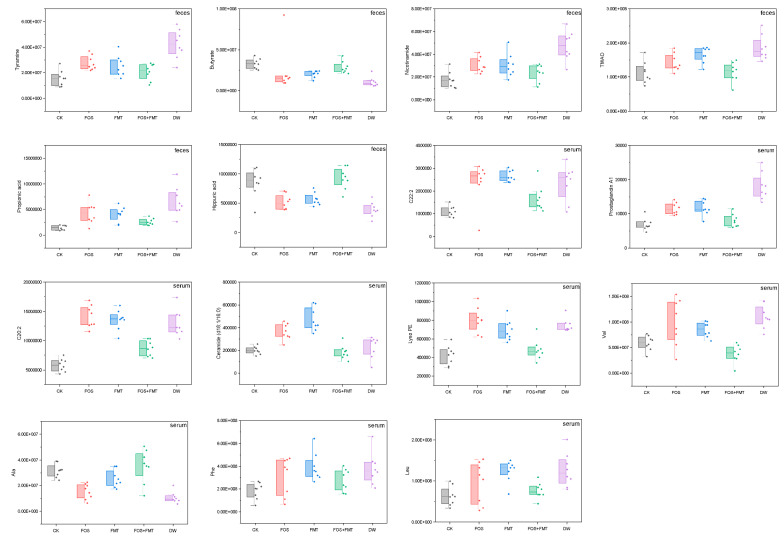
Alterations to metabolites of gut flora and serum after intervention to mice circulation (*n* = 8).

**Figure 8:**
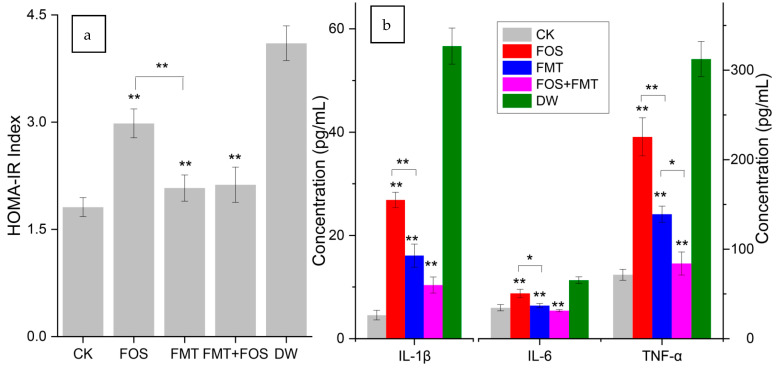
Effects of the interventions on host physiology: (**a**) IR condition and (**b**) inflammation condition (*n* = 4). Data are expressed as the mean ± SEM (* *p* < 0.05; ** *p* < 0.01), and the basis of significance analysis was the DW group.

Significant differences of IR were obviously obtained between the exposure group and the dietary treatment group (Figure 7 and Figure 8). The IR index was remarkably lowered by FOS and FMT, and both of them were effective means to improve IR (Figure 8a), as reported in the literature [35]. FMT was more effective than FOS in improving mouse IR caused by pesticide exposure, and the insulin sensitivity was basically reverted to a normal level. The FOS and FMT group presented similar effects with that in single treatment of FMT, indicating that FMT played a primary role in improving IR. Rapidly responding inflammatory cytokines in the serum, namely, IL-1β, IL-6, and TNF-α, were observed after the treatments, as shown in Figure 8b. The contents of cytokines significantly decreased in the serum, indicating that host inflammatory status was remarkably released. The effects of these treatments suggest that the combined treatments of FOS and FMT enhanced the effects of single-factor treatments, and levels of IL-6 and TNF-α almost returned to their initial.

The contents of some metabolites in the gut and serum were observed. Butyrate maintained host fullness by stimulating the vagus nerve and promoted fat oxidation to restrict host diet. A high content of butyrate prevented diet-induced obesity and increased insulin sensitivity. Pesticide exposure obviously decreased the butyrate content in the gut, while the use of FOS, FMT, and FOS and FMT enhanced it, with the FOS and FMT treatment being the most effective. Propionic acid is effective in decreasing host cholesterol, relieving hypertension, relieving inflammation, and reducing liver fat. However, in the present work, the exposed group showed an increased propionic acid concentration in the feces, and the mechanism was not found. Overall, pesticide exposure altered the intestinal flora and their SCFAs, thus increasing the risk of metabolic syndrome in the host. In this work, TMAO had a high positive relationship with leucine in host serum, and they were enhanced by exposure and then decreased by interventions. Moreover, unsaturated fat acids, such as C20:2 and C22:2, also remarkably decreased in the intervention groups. High contents of branched chain amino acids (BCAAs) and aromatic amino acids (AAAs) in the exposure group were remarkably reduced by the treatments, especially by the FOS and FMT treatment, which lowered the risk of T2D. Ceramide was reported to be related to cerebral vascular diseases, IR, and HbA1c (a type of glycated hemoglobin) abnormality; as such, ceramide may be a new biomarker of adverse cardiovascular events [36]. Lyso PE (phosphatidylethanolamine) induces inflammation and increases oxidative stress [30]. They were both the unfavorable factors in the serum increased by pesticide stress, while the conditions were improved in the intervention.

## 4. Discussions

### 4.1. Effects of Pesticide Exposure on Physiological Phenotype

More attention is often paid to the metabolic syndromes caused by mental stress and unhealthy diet [37] than contamination, and the latter is usually underestimated for its characteristics of low concentration and concealments [3] (especially dietary pesticide residue) because it is usually directly taken into the circulating system along with the diet. Many environmental contaminations have been reported to cause obesity or T2D to the human body. For example, dietary chlorpyrifos residue has been found to cause IR and obesity.

Obesity is one of the most common manifestations of metabolic syndrome. In this work, obesity and IR were yielded when the mice were exposed to acetamiprid and tebuconazole. This finding indicated that the chronic dietary pesticide residues of acetamiprid and tebuconazole affected glucose and fat metabolism, although they were low-toxicity and low-level. Further, combination exposure aggravated the intervention to the body’s physiological metabolism. A serum biochemistry analysis showed pesticide exposure may lead to the risk of NAFLD and arteriosclerosis. Observations of inflammatory factors in the serum showed that the body was in a state of chronic inflammation when the mice were exposed to the pesticide. Host tissue histology proved that dietary pesticide exposure lead to NAFLD and leaky gut in the host. The metabolism health risks of chronic dietary pesticide exposure were confirmed.

Avoiding the action target existing in higher animals, especially mammals, is one of the main objectives in designing low-toxicity pesticides. Therefore, we speculate that the effects of dietary pesticides on the body are indirect. Pesticides caused alterations to the gut flora and then affected physiological activity. The intestine is the highest density distribution part of a body’s microbial community and a main source of endogenous LPS [16]. Pesticide exposure caused the accumulation of LPS in the serum and then inflammation to the body. An observation of intestinal permeability indicated that inflammation damaged the intestinal barrier and accelerated the leakage of LPS from the gut to the blood. The decreased intestinal barrier in turn increased inflammation. Therefore, the results in this work proved that the gut flora comprise a significant action pathway of pesticide exposure to host metabolism health that is often neglected due to the low toxicity and residue level of pesticides. The long-term intake of dietary pesticide residue still poses a great threat to body metabolism health, although it is a long process.

### 4.2. Relationships Among Gut Bacteria, Host and Metabolic Disease Risk

The results of the gut flora analysis showed that the inter-population diversity of the gut flora was affected by pesticide exposure, directly proving that action target of the dietary pesticide exposure in the intestinal microflora. Tebuconazole had more effects on the body than acetamiprid. A high ratio of Firmicutes/Bacteroidetes induced host obesity because more Firmicutes made the host obese by releasing signals to the host to transform more blood glucose into fat [18]. The relationships between gut flora and their metabolites were analyzed on the basis of the alterations caused by pesticide exposure. The dominant population of the intestinal microflora, *Alistipes*, *Limnobacter*, *Brevundimonas*, *Enterococcus*, and *Blautia*, was involved, and the compounds used in the analysis were all significantly different metabolites with high correlation coefficients that elevated the relevance of the results. Relevance analysis was an effective mean to present the combined approaches in directional intervention to the gut flora, especially in such complex system. Clarifying the relevance was helpful to confirm the intervention target.

On the basis of bacterial size, multiple intestinal barriers, and host immunity, the gut flora could not easily breakthrough the intestinal barrier and directly intervene the host physiological activity in general. In contrast, the metabolites of the intestinal microflora could easily get into the blood, and they are the main media or the link of dialogue between the gut flora and the host. The compounds produced by the intestinal microflora could get into the blood and be taken to the rest of the body, where they could directly or indirectly participate in host physiological metabolism [38,39].

As the largest digestive gland and main metabolic organ in the body, the liver leads and participates in most substance and energy metabolisms, thus involving great amounts of metabolism pathways. Therefore, investigating the metabolic profiling alterations of liver is an essential way to clarify the health risk mechanisms of pesticide residues. Many vital disordered metabolic pathways were clarified via metabolic profiling, which explained the mechanism of interactions and perfected the research on the process of the health risk caused by pesticides. The findings were theoretical bases for the presentation of treatments or interventions to body metabolic problems.

### 4.3. Effects of Interventions on Stressed Host

As a type of soluble dietary fiber, FOS is not digested by mammalian endogenous enzymes and not absorbed by the small intestine. It is an effective proliferation factor for gut flora and is utilized by many intestinal floras, with the function of regulating host metabolisms, improving health conditions, and mitigating many diseases. In FMT, gut bacteria and their metabolites were found to be the main contents in the used water extract of feces, and they affected the physiological activities flora community [35]. An apparent release to disorder of host metabolism was yielded after interventions, and it proved that FMT and FOS all directly affected host metabolism by intervening the gut. The inflammatory status improved, and the risk metabolites in the serum decreased. FOS improved the disorder of the gut flora through the effects of proliferation on the beneficial bacteria, which rely on dietary fiber, while FMT directly changed the gut flora of the stressed mice via transplantation. The effects of the FOS and FMT treatment were the best, indicating that the transplanted intestinal microflora and supplementary dietary fiber exerted enhanced promotion on the gut flora. FOS and FMT presneted strong evidence that proved that alterations to the gut flora are vital inducements and even sources of health risk caused by long-term dietary exposure to low levels of pesticide residues. The study also supplied clues and insights for treatments or interventions for body metabolic problems on the basis of gut flora.

## 5. Conclusions

In the present study, physiological phenotype and biochemical analyses indicated that chronic metabolic syndrome, IR, gut leak, NAFLD, and obesity occurred in mice. Starting from the clue of metabolic disorders, the reasons were found in the gut flora. Then, the alterations in gut flora and their metabolism under exposure were observed. Many harmful intestinal microflora and risk factors of body metabolism were found, and their correlations were determined. The effects of pesticide exposure on host physiological activities were characterized via metabolic profiling. Considering the role of metabolites in the reaction between the intestinal microflora and the host, the correlations between the metabolites of gut flora and host were explored. Several altered metabolic pathways of host circulation were identified in accordance with the different metabolites in the liver. Finally, the intervention experiment verified that the affected gut flora comprised the vital medium and inducement in the metabolic health risk caused by pesticide exposure. This work showed that the gut flora comprised the action pathway of the host metabolic health risk caused by long-term exposure to low levels dietary pesticide residues. The findings served as theoretical bases for the presentation of treatments or interventions to body metabolic problems. This work also supplied clues and insights for the treatment or intervention of body metabolic problems on the basis of gut flora.

## Data Availability

Data is contained within the article or supplementary material.

## Supplementary Materials

The following are available online at https://www.mdpi.com/article/10.3390/foods10040835/s1.
