# Supplementary material for: Gut Flora-Mediated Metabolic Health, the Risk Produced by Dietary Exposure to Acetamiprid and Tebuconazole
Source: Foods. 2021 Apr 12;10(4):835. doi: 10.3390/foods10040835 (PMC8070257; doi:10.3390/foods10040835)
Supplement: Supplementary file 1 [file foods-10-00835-s001.zip › supplementary files/Supplementary File 1ú║M1.pdf]

Detailed and Methods for Untargeted Metabolomics.

2.9. Untargeted and targeted Metabolic Profiling Analysis

Untargeted metabolomics analysis: Polar and low-polar solvents were used in the analyte extraction procedure. For the liquid samples, four times the volume of methanol (MeOH): acetonitrile (ACN) (v: v = 1:1) was added into the serum or urine samples. Then, the mixture was blended for 1 min, incubated for 10 min at −20 °C, and concentrated at 13000 g and 0 °C. Subsequently, the supernatant was filtered through 0.22 μm membranes to yield the polar extraction solution of the serum or urine samples. The system methyl tert-butyl ether: MeOH: sample (serum or urine) = 10:2:5 was employed in low-polar analyte extraction. After the same pretreatments and centrifugation were conducted, the supernatant was transferred into a new tube, blown dry with nitrogen, and reconstituted with CH_3Cl: MeOH = 1:3. The liver tissue was homogenized with pre-chilled MeOH: ACN: water (v: v = 2:2:1, 0 °C) in accordance with the proportion at 100 mg/mL. The slurry was incubated for 30 min at −20 °C and concentrated at 13000 g and 0 ℃. A similar volume of the supernatant for each sample was transferred into a new centrifugal tube and concentrated into powder by nitrogen blowing and vacuum freezing. A polar extraction solution of the liver was prepared by reconstituting the powder with MeOH: water (v: v = 4:1) and filtering through 0.22 μm membranes. The precipitate of the liver samples was suspended with pre-chilled MeOH: CH_2Cl_2 (v: v = 1:3, 0 ℃) in accordance with the same proportion. Then, the remaining procedures were repeated in accordance with that in the polar extract, and this is the pretreatment for low-polar extract of liver. For different moisture contents, the mouse feces were dried by vacuum freezing. The pretreated feces were immersed in pre-chilled MeOH: ACN: water (v/v = 2:2:1, 0 °C) at 100 mg/mL and −20 °C for 1 hour and subjected to sonication for 15 min on an ice bath. Then, polar or non-polar extracts were obtained using the same steps in the liver samples.

All the extracted samples were analyzed using high-performance liquid chromatography (HPLC)-HRMS (QE plus, Thermo Scientific) operating with BEH T3 (Waters, USA) column for the separation of polar and low-polar extracts and BEH HILIC for the polar extract. The positive and negative scan modes were separately applied for every sample. The MS parameters (electrospray ionization, ESI) were optimized and set as follows: spray voltage, 4 kV for positive mode and 3.5kV for negative mode; sheath gas flow rate (N_2), 40 arbitrary units (a. u.); auxiliary gas flow rate (N_2), 10 a. u.; and capillary temperature, 300 ℃. Data were acquired in data-dependent analysis mode (Full MS/dd-MS^2), with resolutions of 70,000 for Full MS and 17,500 for dd-MS^2. Monovalent ions with the NC (Nuclear/Cytoplasmic) in 70–1000 and AGC target of more than 10^5 were scanned in the NCE mode (15, 30, and 45). The LC methods for different samples are described in following Table.

Targeted metabolomics analysis: Analysis was performed via ultra-HPLC coupled to an MS/MS detector (QTrap, 6500, AB Sciex) with the ESI source in positive ion mode. A Waters BEH T3 column (1.7 μm, 2.1 × 100 mm) was used for targeted compound separation, with a flow rate of 0.3 mL/min at 35 ℃. The methods were shown in following Table. Multiple reaction monitoring was used to monitor the compounds. The mass parameters were as follows: nebulizing gas flow, 3 L/min; heating gas flow, 10 L/min, interface temperature, 300 ℃; DL temperature, 250 ℃; heat block temperature, 400 ℃; and drying gas flow, 10 L/min. All the amino compound standards were purchased from Sigma-Aldrich (USA).

LC methods for metabolism analysis

**Extract**	**Column**	**Scan mode.**	**Mobile phase A**	**Mobile phase B**	**Elution gradients (A+B=100%)**
**relatively strong polar solution**	HSS T3	+	acetonitrile （with 0.05% formic acid）	water （with 0.05% formic acid）	Mobile phase A：0-0.5min, 3%；0.5-8min, 3%-70%；8-13min, 70%-85%；13-13.1min, 85%-100%；13.1-17.5min, 100%；17.5-17.6min, 100%-3%；17.6-21min, 3%
		-	acetonitrile	water （with 5 mM ammonium formate，pH=8.5）	Mobile phase A：0-0.5min, 3%；0.5-8min, 3%-70%；8-15min, 70%-90%；15-15.1min, 90%-100%；15.1-18min, 100%；18-18.1min, 100%-3%；18.1-21min, 3%
	HILIC	+	acetonitrile: water=95:5 (with 5 mM ammonium acetate +0.1% formic acid)	acetonitrile: water=1:1 (with 5 mM ammonium acetate +0.1% formic acid)	Mobile phase A：0-0.5min, 95%；0.5-12min, 95%-75%；12-12.1min, 75%-95%；12.1-15min, 95%
		-	acetonitrile: water=95:5 (with 5 mM ammonium acetate，pH=8.5)	acetonitrile: water=1:1 (with 5 mM ammonium acetate，pH=8.5)	Mobile phase A：0-0.5min, 95%；0.5-12min, 95%-75%；12-14min, 75%；14-14.1min, 95%；14.1-17min, 95%
**relatively low polar solution**	HSS T3	+	acetonitrile (with 0.05% formic acid)	water (with 0.05% formic acid)	Mobile phase A：0-1min, 20%；1-3min, 20%-70%；3-8min, 70%；8-8.1min, 70%-20%；8.1-11min, 20%
		-	acetonitrile	water (with 5 mM ammonium formate，pH=8.5)	Mobile phase A：0-0.5min, 20%；0.5-2min, 20%-70%；2.5-7min, 70%-80%；7-7.1min, 80%-100%；7.1-10min, 100%；10-10.1min, 100%-20%；10.1-13min，20%

LC/MS methods for metabolites detection

(*, quantitative ion pair)

**Medium**	**Compounds**	**Scan mode**	**ion pairs**	**DP (V)**	**CE (V)**	**Mobile phase A**	**Mobile phase B**	**Elution gradients (A+B=100%)**
feces	tyramine	+	138.2/77*	15	35	water（with 5 mM Ammonium Formate , pH=4）	acetonitrile（with 5 mM Ammonium Formate , pH=4）	Mobile phase A：0-4min, 95%-80%; 4-4.1min, 80%-95%; 4.1-6min, 95%
			138.2/91		31			
	butyrate	-	87/87*	-10	-8			
	nicotinamide	+	121/103*	60	21			
			121/93		20			
	TMAO	+	76/58.2*	45	22			
	propionate	-	73/73*	-5	-5			
	hippuric acid	-	178/134*	-15	-15			
Serum	C22:2	-	335/335*	-25	-10	water（with 5 mM Ammonium Formate , pH=8.5）	acetonitrile（with 5 mM Ammonium Formate , pH=8.5）	Mobile phase A：0-5min, 30%-0%; 5-10min, 0%; 10-11.1min, 0-30%; 11.1-12min, 30%;
	C20:2	-	307/307*	-25	-18			
	prostaglandin A	-	335/335*	-50	-10			
			335/317*		-15			
	ceramide (d18:1/16:0)	+	546/264*	120	45			
	Lyso PE	+	464/292*	50	35			
			464/310		27			
	Val	+	118/72*	55	11	water（with 0.05% Formic acid）	acetonitrile（with 0.05% Formic acid）	Mobile phase A：0-3min, 90%-60%; 3-3.1min, 60%-90%; 3.1-5min, 90%
			118/55	26	26			
	Ala	+	90/44*	40	16			
	Phe	+	166/120*	35	14			
			166/103	40	12			
	Leu	+	166/86*	24	12			
			166/69	41	17			

Sequencing method

1. Extraction of genome DNA

Total genome DNA from samples was extracted using CTAB/SDS method. DNA concentration and purity was monitored on 1% agarose gels. According to the concentration, DNA was diluted to 1ng/μl using sterile water.

2. Amplicon Generation

Primer: 16S V4: 515F-806R, 18S V4: 528F-706R, 18S V9: 1380F-1510R, ITS1: ITS5-1737F、ITS2-2043R，ITS2：ITS3-2024F、ITS4-2409R.

16S /18S rRNA genes were amplified used the specific primer with the barcode. All PCR reactions were carried out in 30μL reactions with 15μL of Phusion® High-Fidelity PCR Master Mix (New England Biolabs); 0.2μM of forward and reverse primers, and about 10 ng template DNA.Thermal cycling consisted of initial denaturation at 98 ^{o}C for 1 min, followed by 30 cycles of denaturation at 98 ^{o}C for 10 s, annealing at 50 ^{o}C for 30 s, and elongation at 72 ^{o}C for 30 s. Finally 72 ^{o}C for 5 min.

3. PCR Products quantification and qualification

Mix same volume of 1X loading buffer (contained SYB green) with PCR products and operate electrophoresis on 2% agarose gel for detection. Samples with bright main strip between 400-450bp were chosen for further experiments.

4. PCR Products Mixing and Purification

PCR products was mixed in equidensity ratios. Then, mixture PCR products was purified with GeneJET Gel Extraction Kit(Thermo Scientific).

5. Library preparation and sequencing

Sequencing libraries were generated using Illumina TruSeq DNA PCR-Free Library Preparation Kit (Illumina, USA) following manufacturer's recommendations and index codes were added. The library quality was assessed on the Qubit@ 2.0 Fluorometer (Thermo Scientific) and Agilent Bioanalyzer 2100 system. At last, the library was sequenced on an Illumina NovaSeq platform and 250 bp paired-end reads were generated.

Data analysis

Paired-end reads from the original DNA fragments are merged by using FLASH[1] -a very fast and accurate analysis tool which is designed to merge paired-end reads when there are overlaps between reads1 and reads2. Paired-end reads was assigned to each sample according to

the unique barcodes.Sequences were analyzed using QIIME[2] software package (Quantitative Insights Into Microbial Ecology), and in-house Perl scripts were used to analyze alpha- (within samples) and beta- (among samples) diversity. First, reads were filtered by QIIME quality filters. Then we use pick_de_novo_otus.py to pick operational taxonomic units (OTUs) by making OTU table. Sequences with ≥97% similarity were assigned to the same OTUs. We pick a representative sequences for each OTU and use the RDP classifier[3] to annotate taxonomic information for each representative sequence. In order to compute Alpha Divesity, we rarify the OTU table and calculate three metrics: Chao1 estimates the species abundance; Observed Species is estimates the amount of unique OTUs found in each sample, and Shannon index. Rarefaction curves were generated based on these three metrics.

QIIME calculates both weighted and unweighted unifrac, which are phylogenetic measures of beta diversity. We used unweighted unifrac for Principal Coordinate Analysis (PCoA) and Unweighted Pair Group Method with Arithmetic mean (UPGMA) Clustering. PCoA helps to get principal coordinates and visualize them from complex, multidimensional data. It takes a transformation from a distance matrix to a new set of orthogonal axes. By which the maximum variation factor is demonstrated by first principal coordinate, and the second maximum one by the second principal coordinate, and so on. UPGMA Clustering is a type of hierarchical clustering method using average linkage and can be used to interpret the distance matrix. To mine deeper data of microbial diversity of the differences between the samples, significance test were conducted with some statistical analysis methods, including T-test、MetaStat、LEfSe、Anosim and MRPP.

References

[1] Magoč T, Salzberg SL. FLASH: fast length adjustment of short reads to improve genome assemblies[J]. Bioinformatics, 2011, 27(21): 2957-2963.

[2] Caporaso J G, Kuczynski J, Stombaugh J, et al. QIIME allows analysis of high-throughput community sequencing data[J]. Nature methods, 2010, 7(5): 335-336.

[3] Wang Q, Garrity G M, Tiedje J M, et al. Naive Bayesian classifier for rapid assignment of rRNA sequences into the new bacterial taxonomy[J]. Applied and environmental microbiology, 2007, 73(16): 5261-5267.
