# Supplementary material for: Gut Flora-Mediated Metabolic Health, the Risk Produced by Dietary Exposure to Acetamiprid and Tebuconazole
Source: Foods. 2021 Apr 12;10(4):835. doi: 10.3390/foods10040835 (PMC8070257; doi:10.3390/foods10040835)
Supplement: Supplementary file 1 [file foods-10-00835-s001.zip › supplementary files/Supplementary File 14ú║Table S3.docx]

Table S5 Alterations to serum metabolites of mice exposed to pesticides

| **Metabolites** | **Fold Change Value** | | | | |
| --- | --- | --- | --- | --- | --- |
|  | D/  CK | W/  CK | DW/  CK | DW/D | DW/W |
| (13Z,16Z)-docosadienoic acid | 5.66 | 6.29 | 11.77 | 2.08 | 1.87 |
| (7E,10E,13E,16E)-7,10,13,16-Docosatetraenoic acid | 1.98 | 1.44 | 2.77 | 1.40 | 1.92 |
| 8Z,11Z,14Z-Eicosatrienoic acid | 1.72 | 2.27 | 2.19 | 1.27 | 0.97 |
| all-cis-4,7,10,13,16-Docosapentaenoic acid | 1.82 | 2.03 | 3.40 | 1.87 | 1.68 |
| Docosatrienoic acid | 2.53 | 2.86 | 5.56 | 2.19 | 1.95 |
| Methyl (6Z,12Z,15Z)-6,12,15-octadecatrienoate | 3.05 | 3.09 | 4.74 | 1.55 | 1.54 |
| 1-nonanoic acid | 4.58 | 2.54 | 2.29 | 0.50 | 0.90 |
| (2E)-2-Nonenoic acid | 2.97 | 2.72 | 2.77 | 0.93 | 1.02 |
| Valine | 1.24 | 1.03 | 1.10 | 0.89 | 1.07 |
| DL-Leucine | 1.86 | 1.19 | 1.43 | 0.77 | 1.19 |
| L-Phenylalanine | 1.26 | 1.66 | 1.96 | 1.55 | 1.18 |
| L-Glutamic acid | 15.57 | 0.85 | 0.58 | 0.04 | 0.69 |
| DL-Citrulline | 2.31 | 1.08 | 1.33 | 0.58 | 1.23 |
| 3-Methylhistidine | 0.34 | 0.33 | 0.50 | 1.48 | 1.52 |
| 5-Amino-D-isoleucine | 0.78 | 0.98 | 1.33 | 1.71 | 1.35 |
| N-Acetyl-L-leucine | 1.14 | 1.50 | 1.40 | 1.23 | 0.93 |
| 3-Methylaspartic acid | 2.43 | 3.22 | 1.97 | 0.81 | 0.61 |
| Aceglutamide | 0.72 | 1.12 | 0.54 | 0.75 | 0.48 |
| 1-Methylhistidine | 0.38 | 0.72 | 0.36 | 0.94 | 0.50 |
| L-(-)-Asparagine | 0.74 | 0.48 | 0.99 | 1.34 | 2.06 |
| L-(-)-Threonine | 0.16 | 0.15 | 0.32 | 2.04 | 2.17 |
| (5R)-5-[(1S)-1,2-Dihydroxyethyl]-alpha-D-lyxopyranose | 2.10 | 1.35 | 1.24 | 0.59 | 0.92 |
| 5-O-alpha-L-Arabinofuranosyl-alpha-L-arabinofuranose | 2.66 | 2.28 | 3.32 | 1.25 | 1.46 |
| Methyl 6-deoxy-2,3-O-isopropylidene-alpha-L-mannopyranoside | 2.77 | 1.29 | 1.52 | 0.55 | 1.18 |
| 1-(1Z-hexadecenyl)-sn-glycero-3-phosphocholine | 0.76 | 1.21 | 1.54 | 2.03 | 1.27 |
| 1-hexadecanoyl-2-(4Z,7Z,10Z,13Z,16Z,19Z-docosahexaenoyl)-sn-glycero-3-phosphocholine | 0.11 | 0.29 | 0.42 | 3.66 | 1.42 |
| PC(18:3(9Z,12Z,15Z)/18:2(9Z,12Z)) | 1.92 | 2.38 | 3.27 | 1.70 | 1.37 |
| 1-hexadecanoyl-sn-glycero-3-phosphoethanolamine | 1.11 | 1.50 | 1.65 | 1.48 | 1.10 |
| 1-stearoyl-sn-glycero-3-phosphoethanolamine | 1.02 | 1.25 | 1.44 | 1.40 | 1.15 |
| 2-[(11Z,14Z)-icosadienoyl]-sn-glycero-3-phosphoethanolamine | 1.24 | 1.24 | 1.28 | 1.04 | 1.04 |
| D-Sphingosine | 0.56 | 0.93 | 1.22 | 2.16 | 1.31 |
| LysoPC(22:4(7Z,10Z,13Z,16Z)) | 0.78 | 1.06 | 1.99 | 2.56 | 1.88 |
| LysoPC(22:5(7Z,10Z,13Z,16Z,19Z)) | 0.92 | 1.42 | 2.40 | 2.59 | 1.69 |
| lysophosphatidylethanolamine (22:6(4Z,7Z,10Z,13Z,16Z,19Z)/0:0) | 0.68 | 0.77 | 0.86 | 1.26 | 1.11 |
| Cholic acid | 0.17 | 0.93 | 0.18 | 1.05 | 0.19 |
| Cytosine | 0.91 | 1.13 | 2.21 | 2.42 | 1.95 |
| Adenosine | 5.94 | 0.88 | 0.68 | 0.11 | 0.78 |
| Uracil | 0.68 | 0.93 | 1.41 | 2.08 | 1.53 |
| Xanthine | 0.55 | 1.34 | 0.31 | 0.57 | 0.23 |
| 7-Methylxanthine | 4.52 | 0.42 | 0.30 | 0.07 | 0.72 |
| 1-methylhypoxanthine | 0.49 | 0.57 | 0.86 | 1.74 | 1.51 |
| 3-Methylhistamine | 0.55 | 0.45 | 0.82 | 1.51 | 1.82 |
| Spermine | 0.39 | 0.38 | 0.74 | 1.91 | 1.94 |
| 9-Methyluric acid | 0.52 | 2.15 | 1.02 | 1.95 | 0.47 |
| Uric acid | 0.76 | 1.85 | 0.92 | 1.22 | 0.50 |
| Prostaglandin A1 | 1.92 | 2.76 | 1.44 | 0.75 | 0.52 |
| Prostaglandin E1 | 1.56 | 2.53 | 1.53 | 0.99 | 0.61 |
| 15-dehydro-prostaglandin E1 | 0.47 | 1.06 | 0.80 | 1.71 | 1.28 |
