# Supplementary material for: Gut Flora-Mediated Metabolic Health, the Risk Produced by Dietary Exposure to Acetamiprid and Tebuconazole
Source: Foods. 2021 Apr 12;10(4):835. doi: 10.3390/foods10040835 (PMC8070257; doi:10.3390/foods10040835)
Supplement: Supplementary file 1 [file foods-10-00835-s001.zip › supplementary files/titles for supplementary 1-18.docx]

**Supplementary File 1: M1** Detailed Methods in Materials and Methods

**Supplementary File 2: Figure S1** Mice bodyweight of different groups during exposure (n=8). CK: control check group, D: acetamiprid treated group, W: tebuconazole treated group, DW (or WD): combination treatment group.

**Supplementary File 3: Figure S2** Mice serum lipid test indexes (n=4). Data are expressed as the mean ± SEM (**p* < 0.05; ***p* < 0.01). CHO: total serum cholesterol, TG: triglyceride, HDL-C: high density lipoprotein cholesterol, LDL-C: low density lipoprotein cholesterol.

**Supplementary File 4: Figure S3** Mice blood biochemistry indexes (liver function) of mice (n=4). Data are expressed as the mean ± SEM (**p* < 0.05; ***p* < 0.01). AST: aspartate aminotransferase, ALT: alanine aminotransferase, ALP: alkaline phosphatase, TP: total protein, ALB: albumin.

**Supplementary File 5: Figure S4** Concentration of LPS in serum (a) and FITC-dextran in urine (b). Data are expressed as the mean ± SEM (**p* < 0.05; ***p* < 0.01).

**Supplementary File 6: Figure S5** Diversity evaluation (a, b) and different species (c, d) of gut flora under pesticides exposure (a: α-diversity, b: NMDS analysis for β-diversity, c: LDA score=3, d: LDA score=4)

**Supplementary File 7: Figure S6** Effects of pesticides on species distribution of mice gut flora at the level of genus (a) and two families of high LPS-yielding gut microbiota (b)

**Supplementary File 8: Figure S7** Heat map of mice gut flora species distribution at the level of genus. (n=4)

**Supplementary File 9: Figure S8** PCA analysis to the metabolites of gut flora

**Supplementary File 10:** Table S1. Significantly different metabolites from gut flora of mice between pesticides exposure and CK.

**Supplementary File 11: Figure S9** PCA analysis to the metabolites of mice serum

**Supplementary File 12: Figure S10** Heatmap of alterations to metabolites in mice serum. (n=4)

**Supplementary File 13:** Table S2. Different metabolites in gut flora of mice between pesticides exposure and CK.

**Supplementary File 14:** Table S3. Alterations to serum metabolites of mice exposed to pesticides

**Supplementary File 15: Figure S11** CIA analysis between metabolites of mice serum and gut flora

**Supplementary File 16:** Table S4. Mice serum metabolites in associations with gut flora and host.

**Supplementary File 17: Figure S12** The influenced metabolic pathways in liver of mice under pesticides exposure

**Supplementary File 18: Figure S13** PCA analysis to the metabolites of mice liver
