# Supplementary figures and images for: Gut Flora-Mediated Metabolic Health, the Risk Produced by Dietary Exposure to Acetamiprid and Tebuconazole
Source: Foods. 2021 Apr 12;10(4):835. doi: 10.3390/foods10040835 (PMC8070257; doi:10.3390/foods10040835)

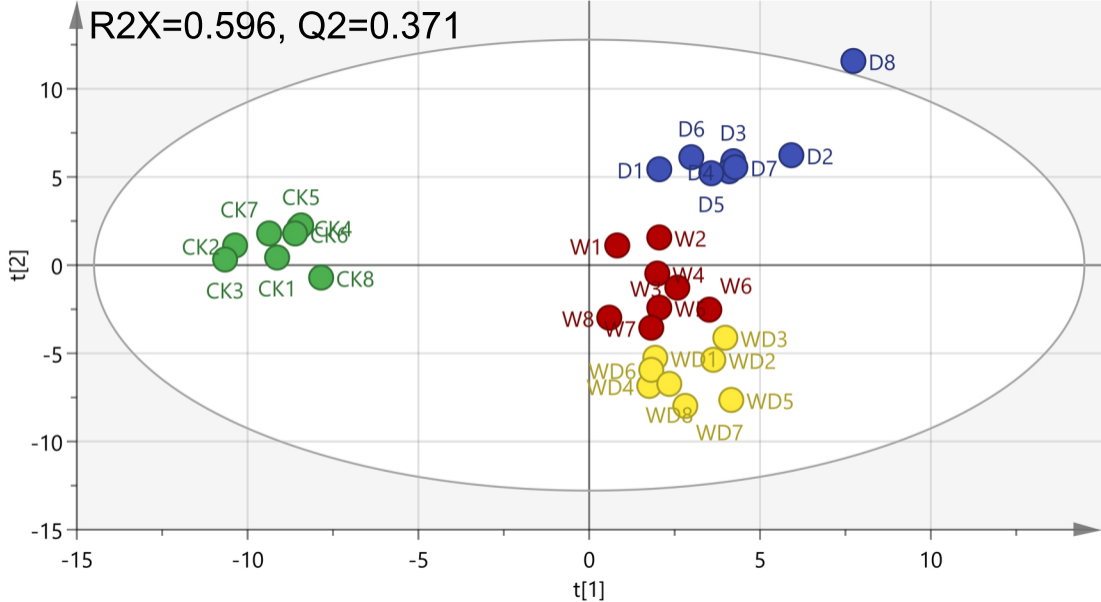

Supplement: Supplementary file 1 [file foods-10-00835-s001.zip › supplementary files/Supplementary File 11ú║Fig. S9.pdf]

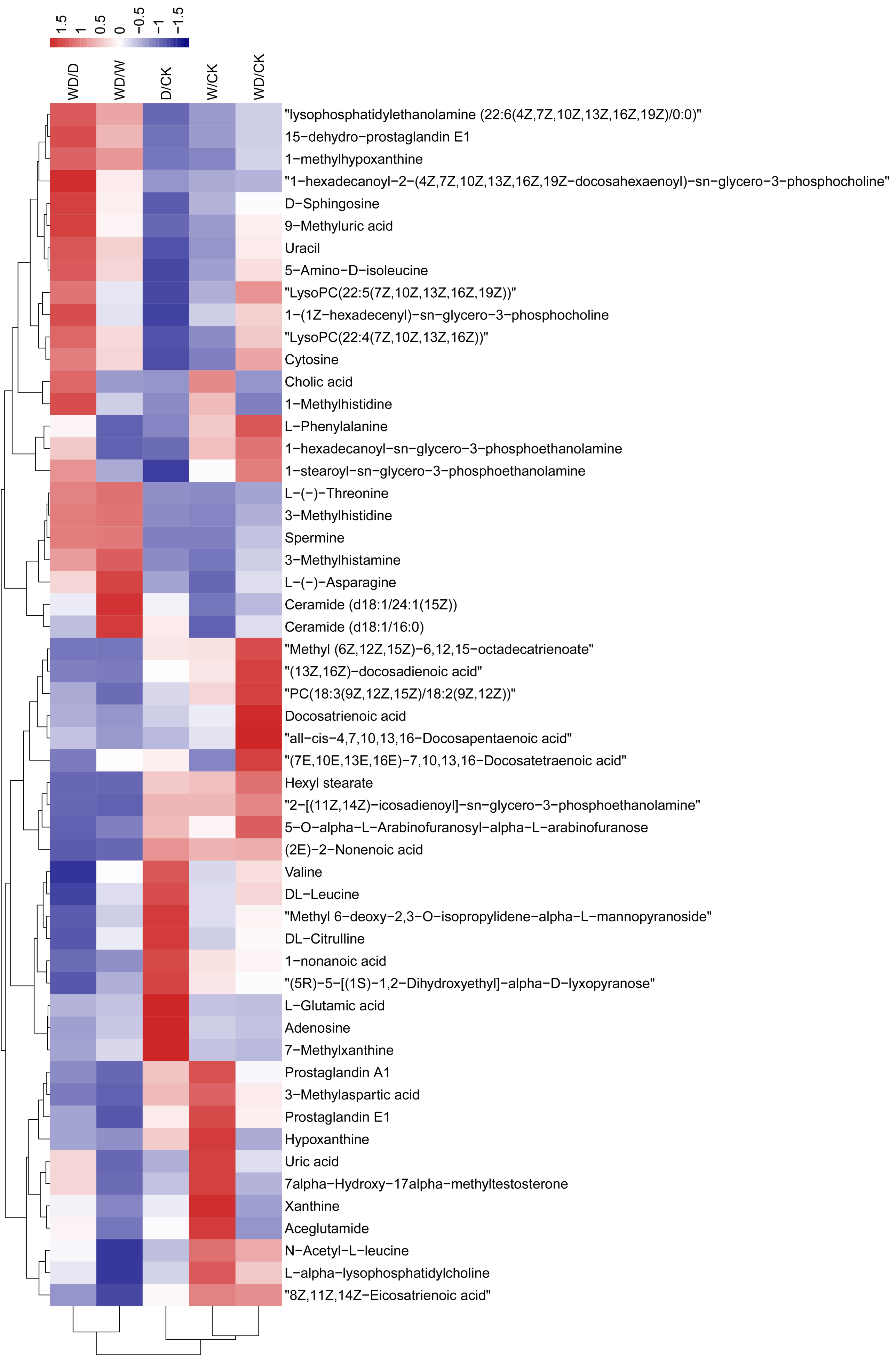

Supplement: Supplementary file 1 [file foods-10-00835-s001.zip › supplementary files/Supplementary File 12ú║Fig. S10.pdf]

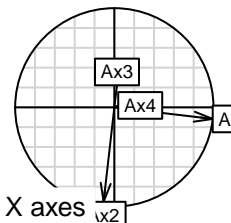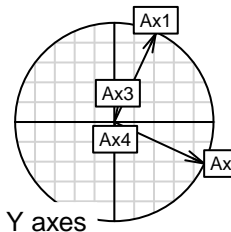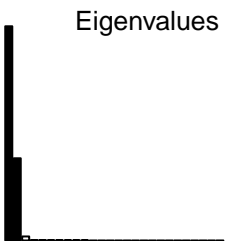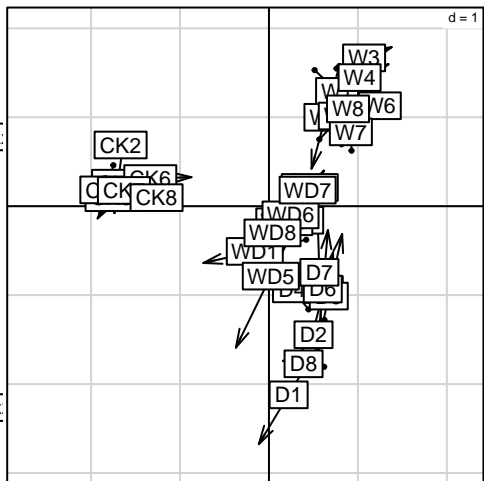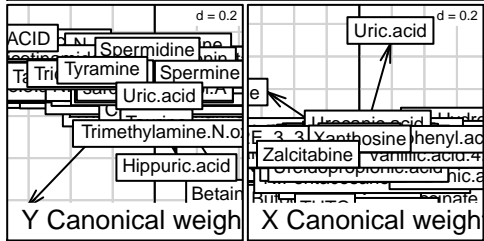

Supplement: Supplementary file 1 [file foods-10-00835-s001.zip › supplementary files/Supplementary File 15ú║Fig. S11.pdf]

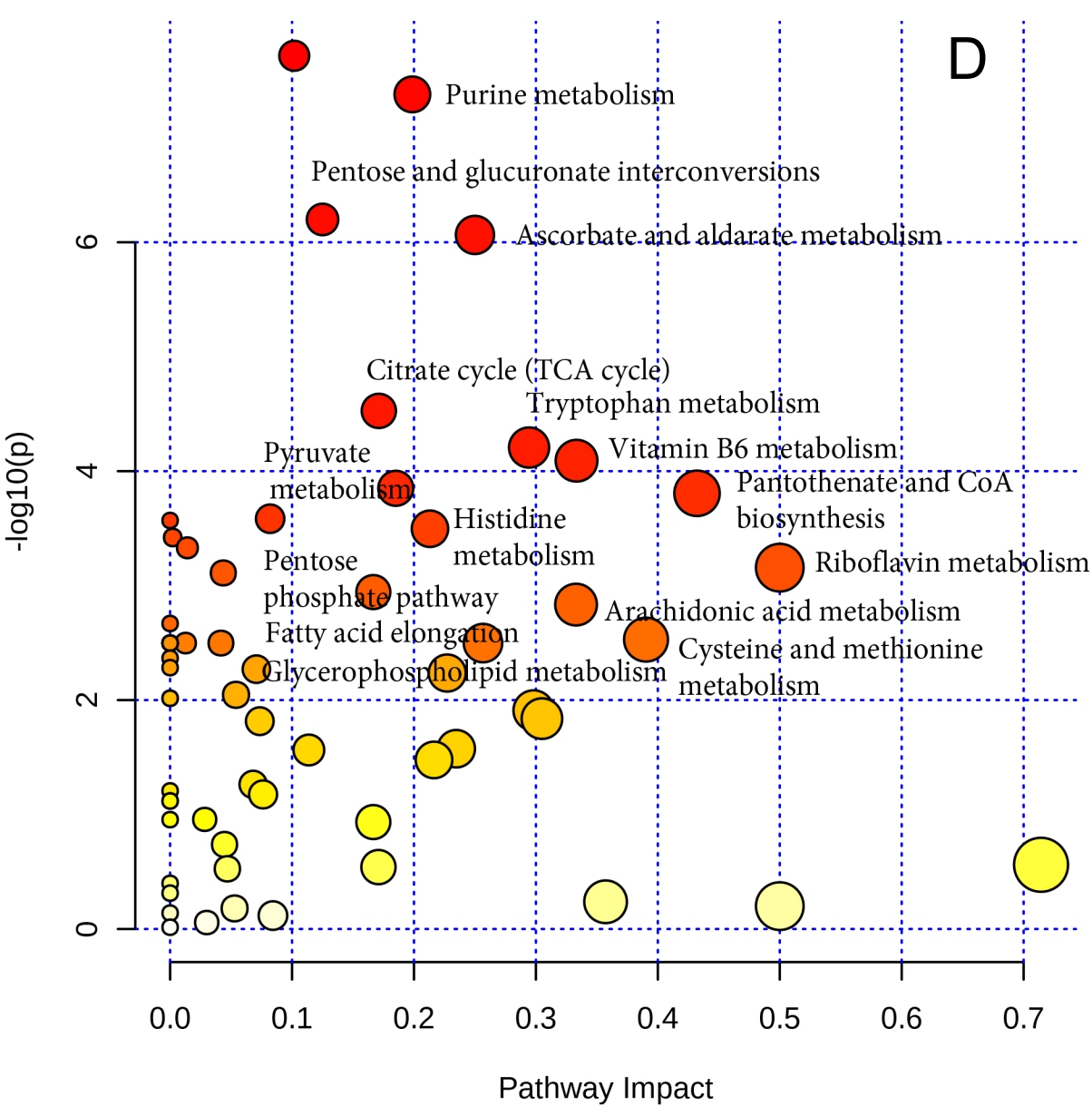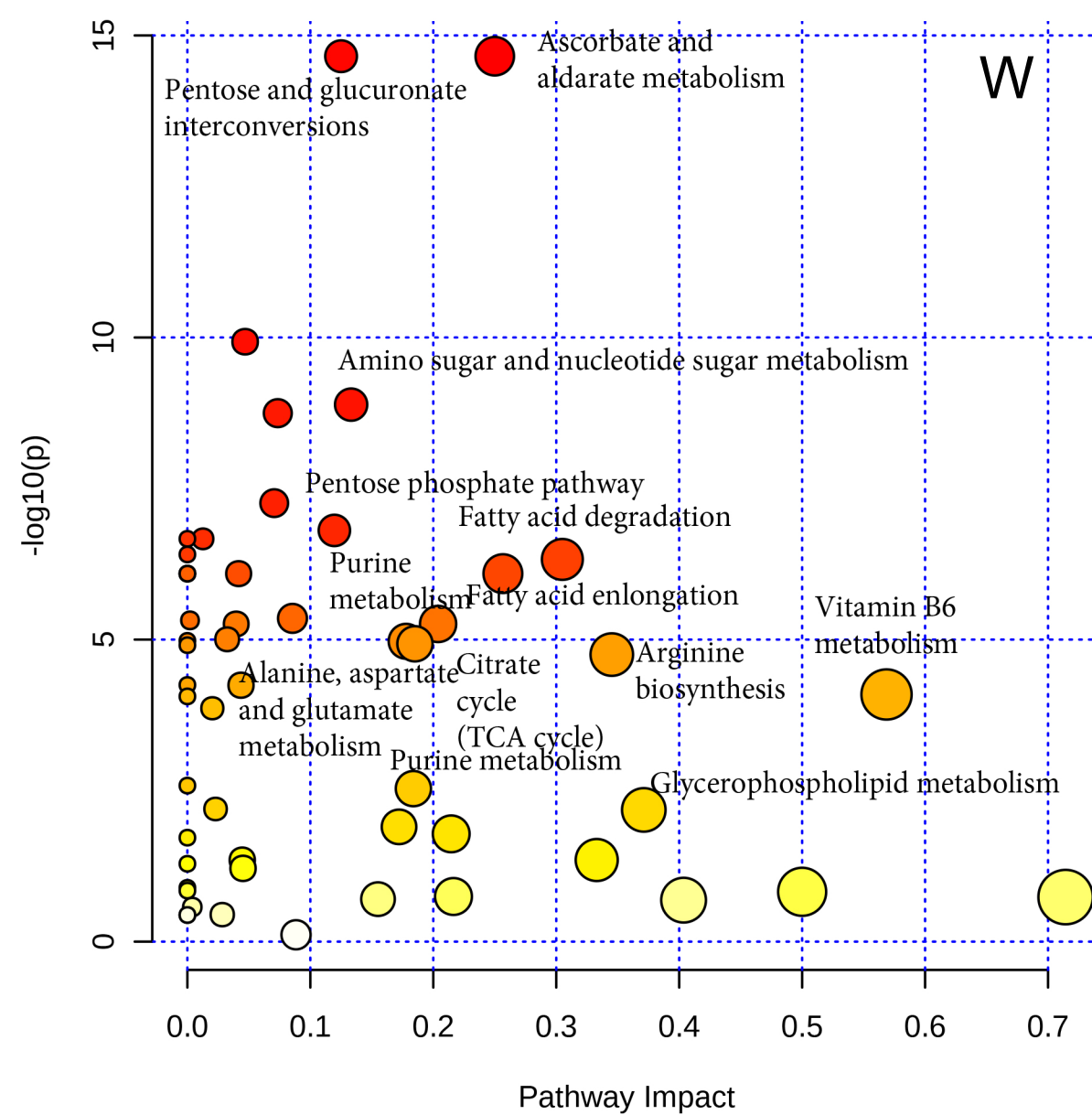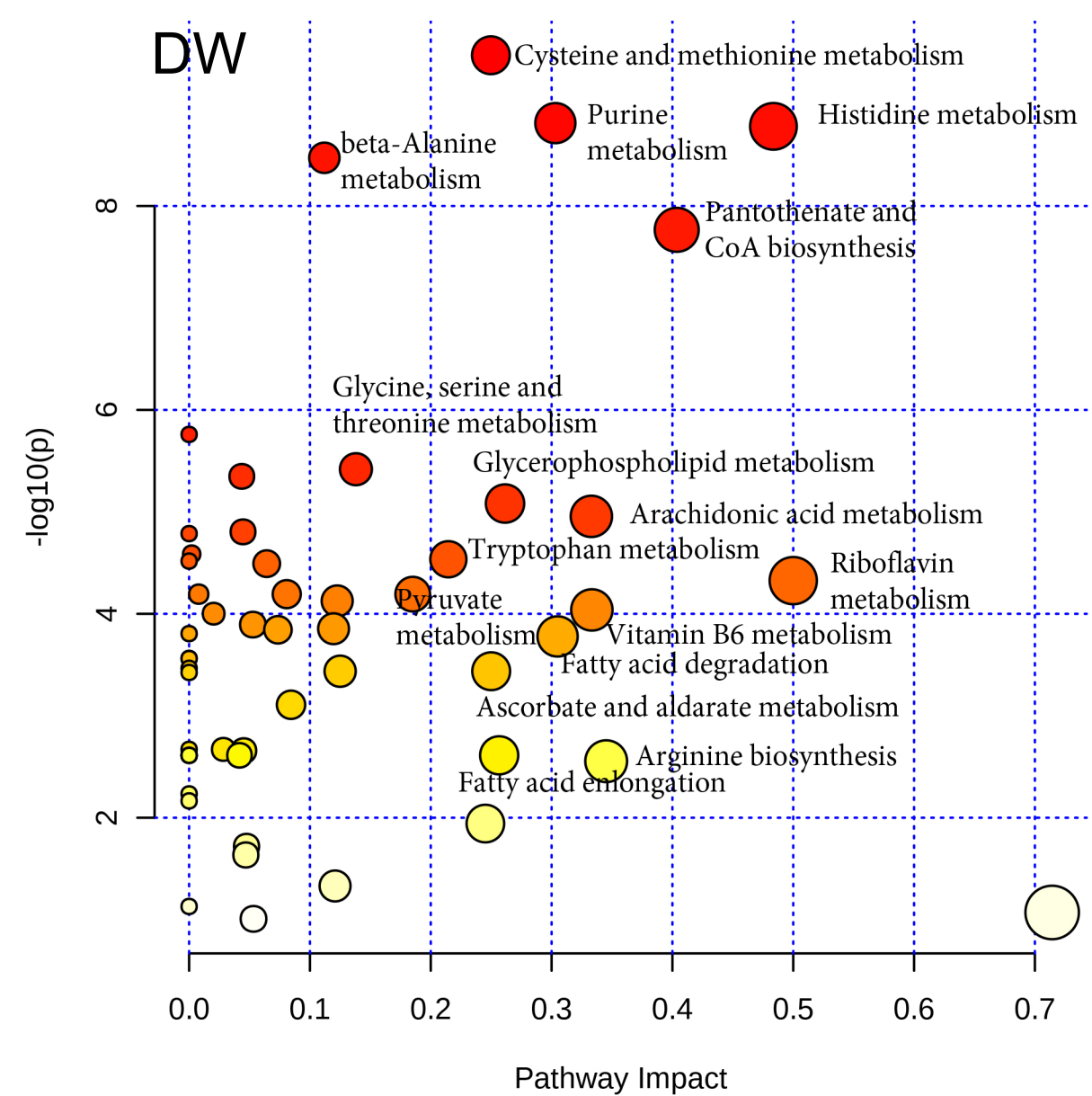

Supplement: Supplementary file 1 [file foods-10-00835-s001.zip › supplementary files/Supplementary File 17ú║Fig. S12.pdf]

$R^2X=0.526$ ,  $Q^2=0.242$

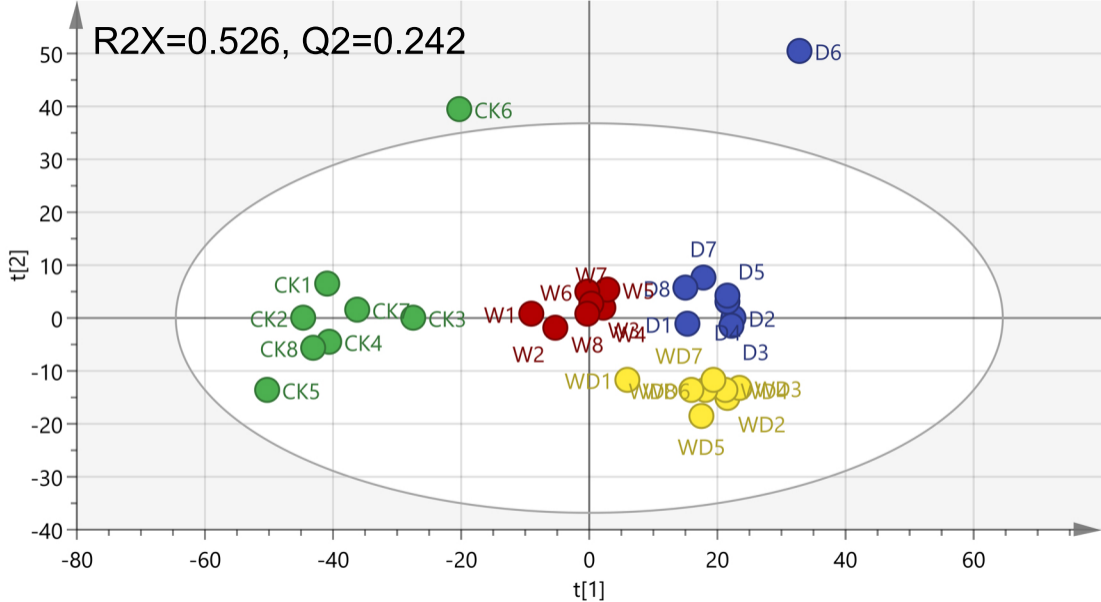

Supplement: Supplementary file 1 [file foods-10-00835-s001.zip › supplementary files/Supplementary File 18ú║Fig. S13.pdf]

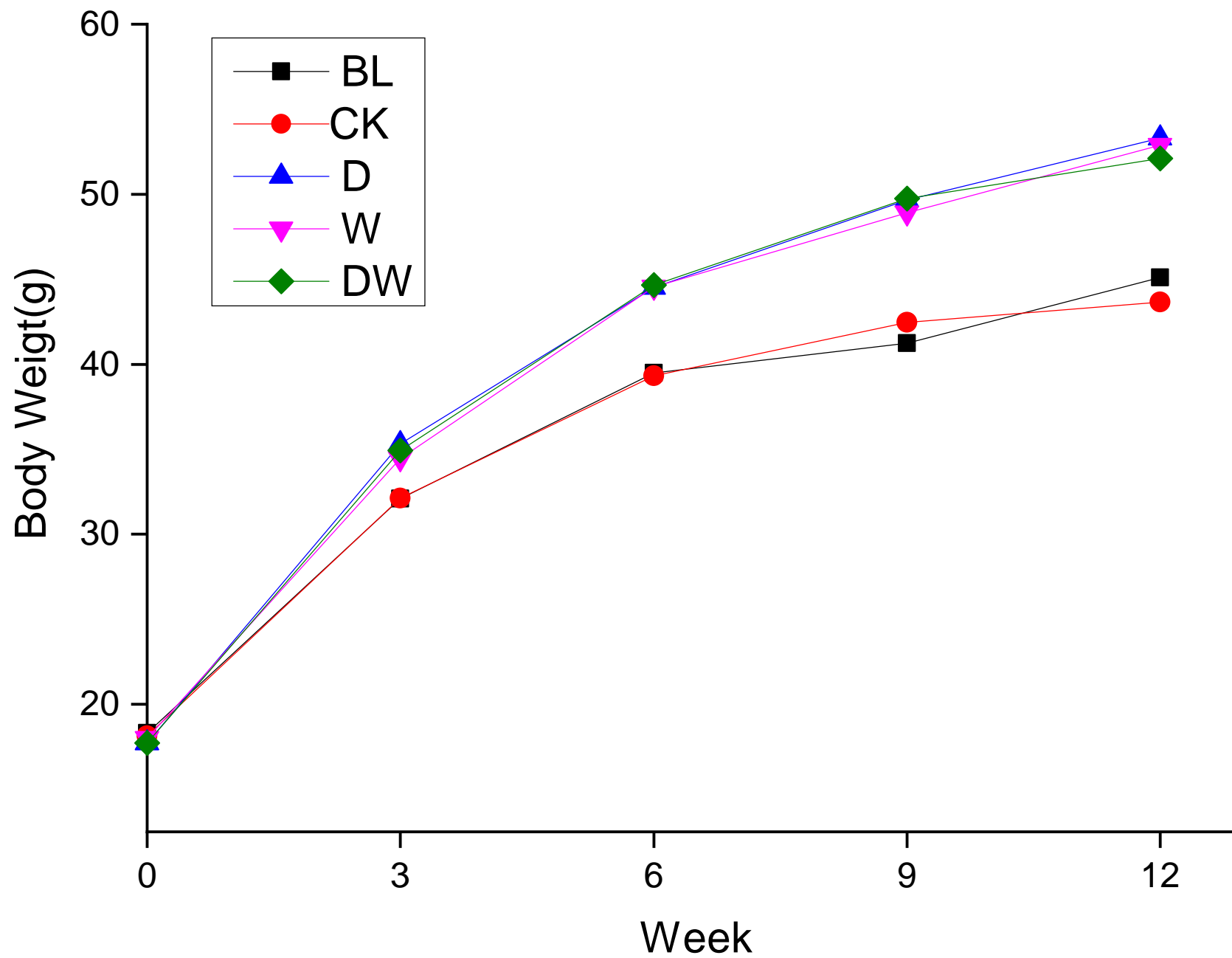

Supplement: Supplementary file 1 [file foods-10-00835-s001.zip › supplementary files/Supplementary File 2ú║Fig. S1.pdf]

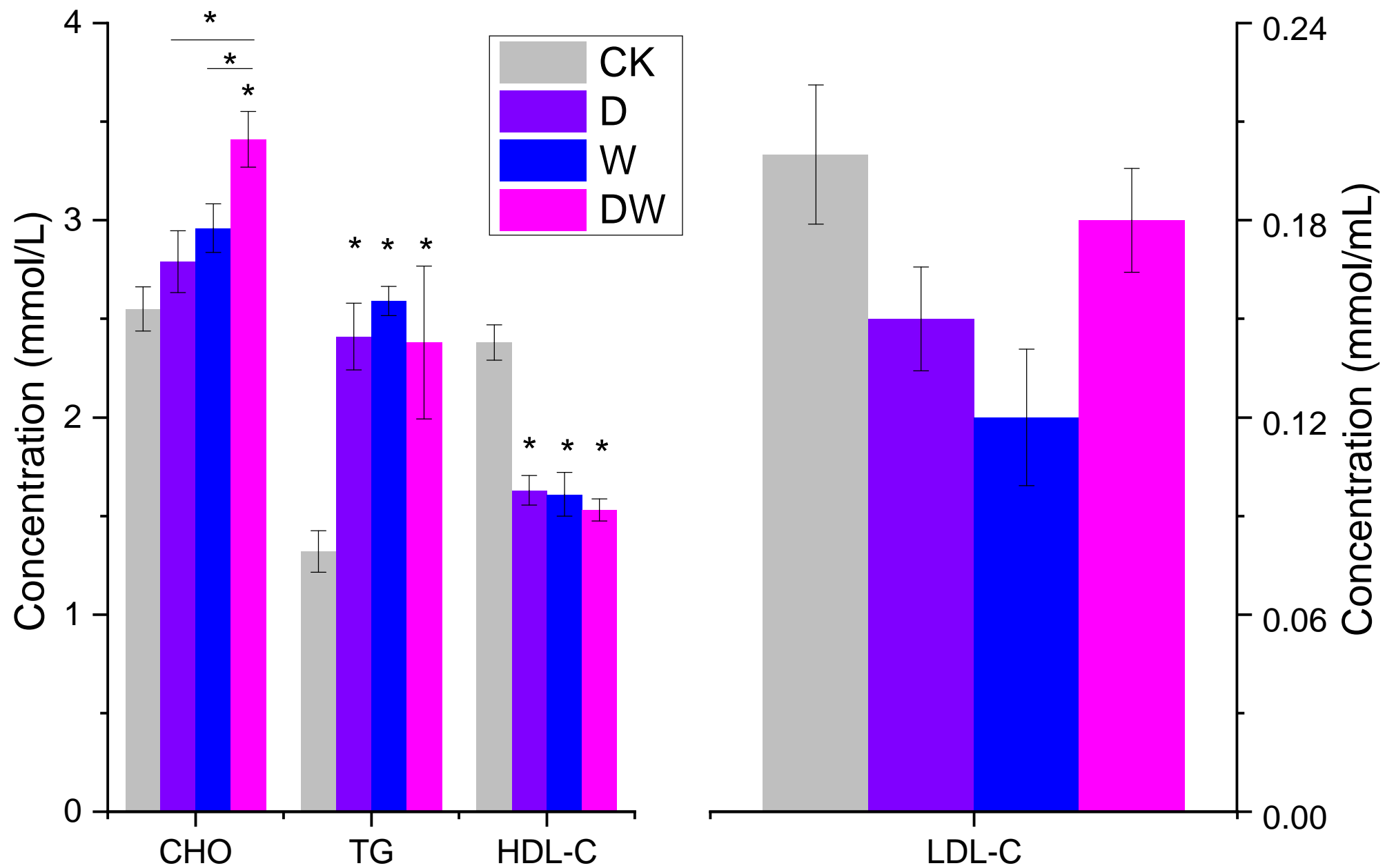

Supplement: Supplementary file 1 [file foods-10-00835-s001.zip › supplementary files/Supplementary File 3ú║Fig. S2.pdf]

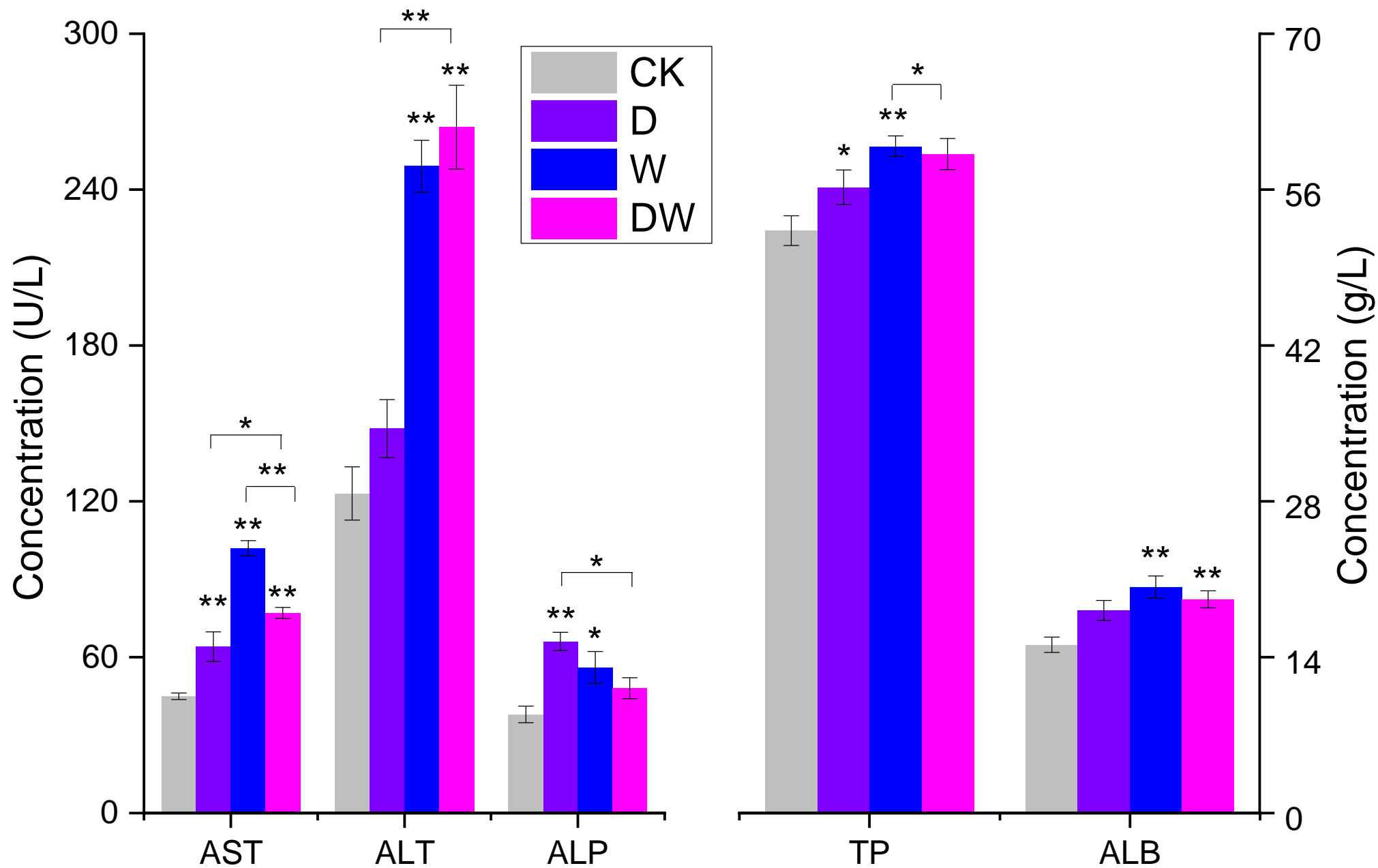

Supplement: Supplementary file 1 [file foods-10-00835-s001.zip › supplementary files/Supplementary File 4ú║Fig. S3.pdf]

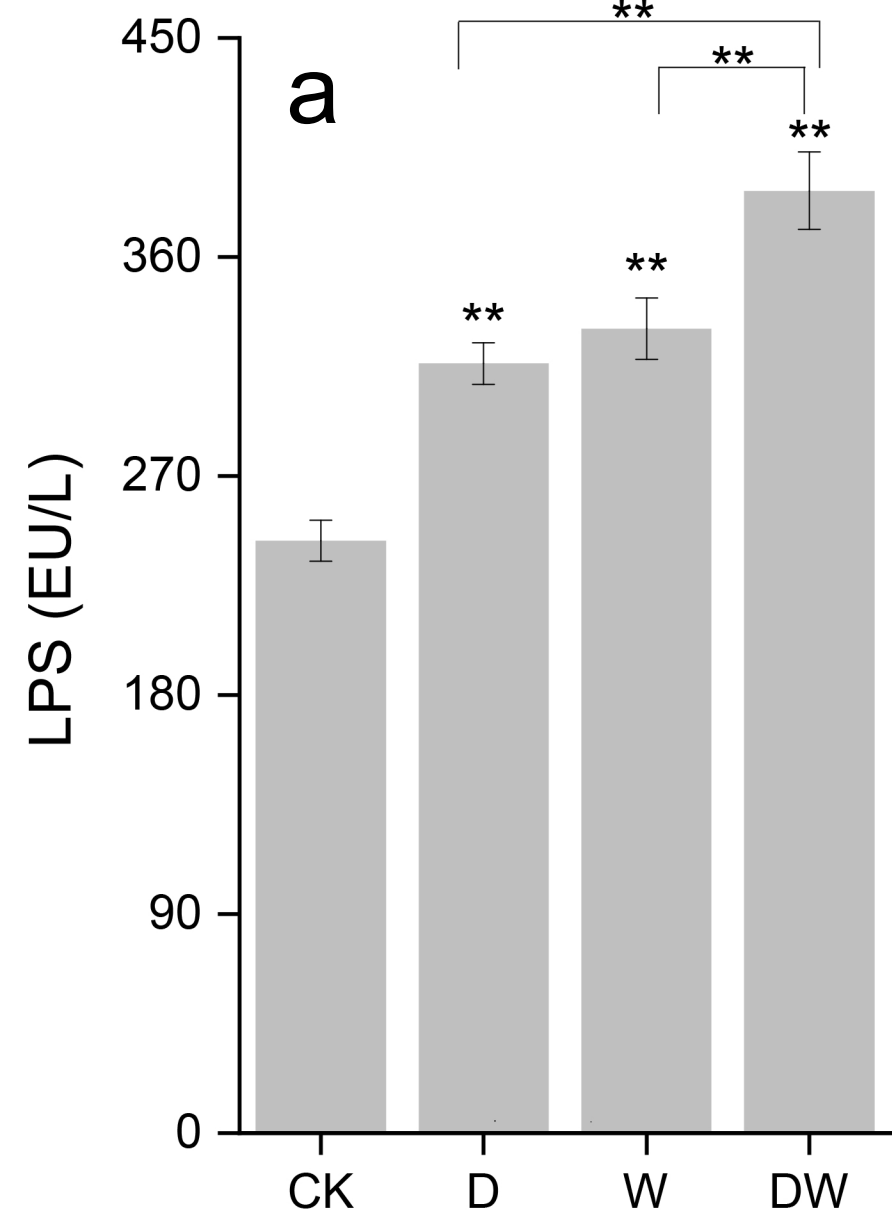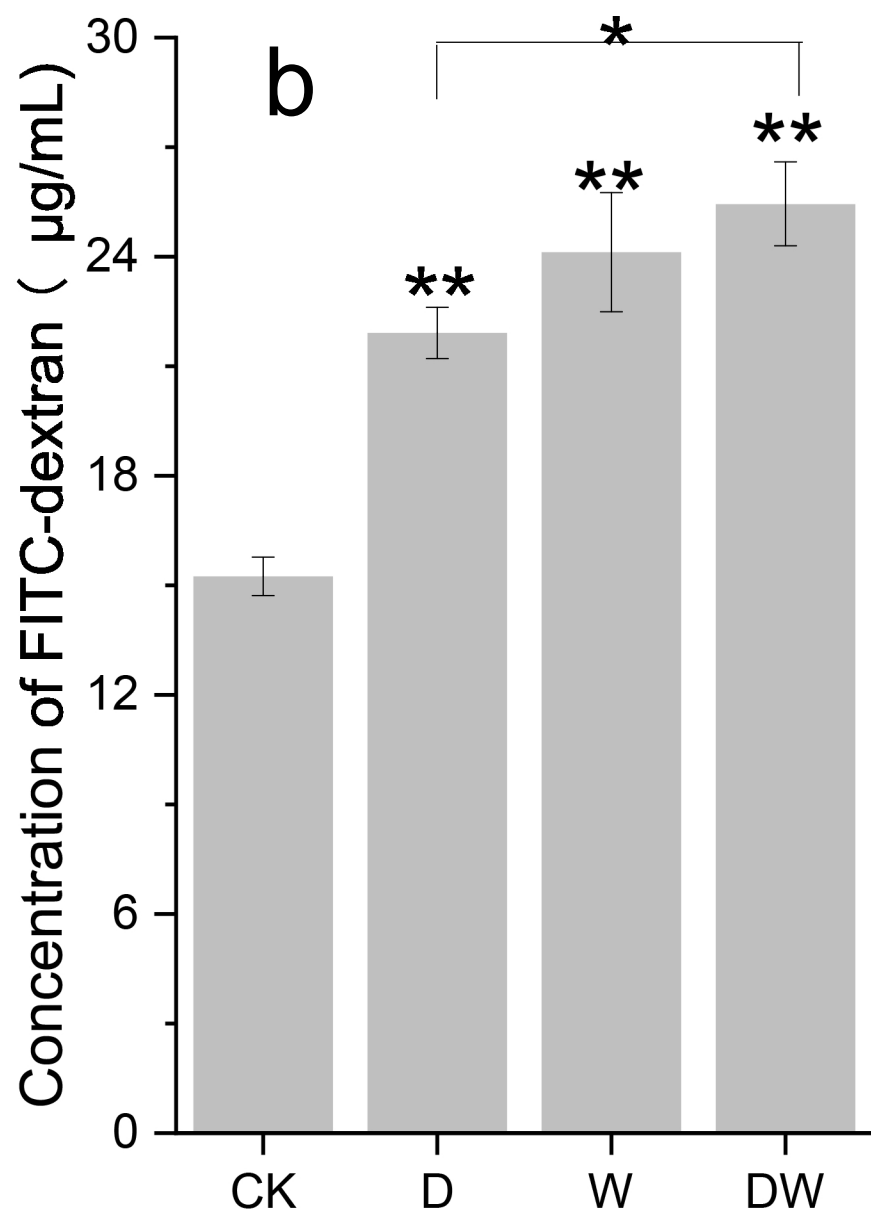

Supplement: Supplementary file 1 [file foods-10-00835-s001.zip › supplementary files/Supplementary File 5ú║Fig. S4.pdf]

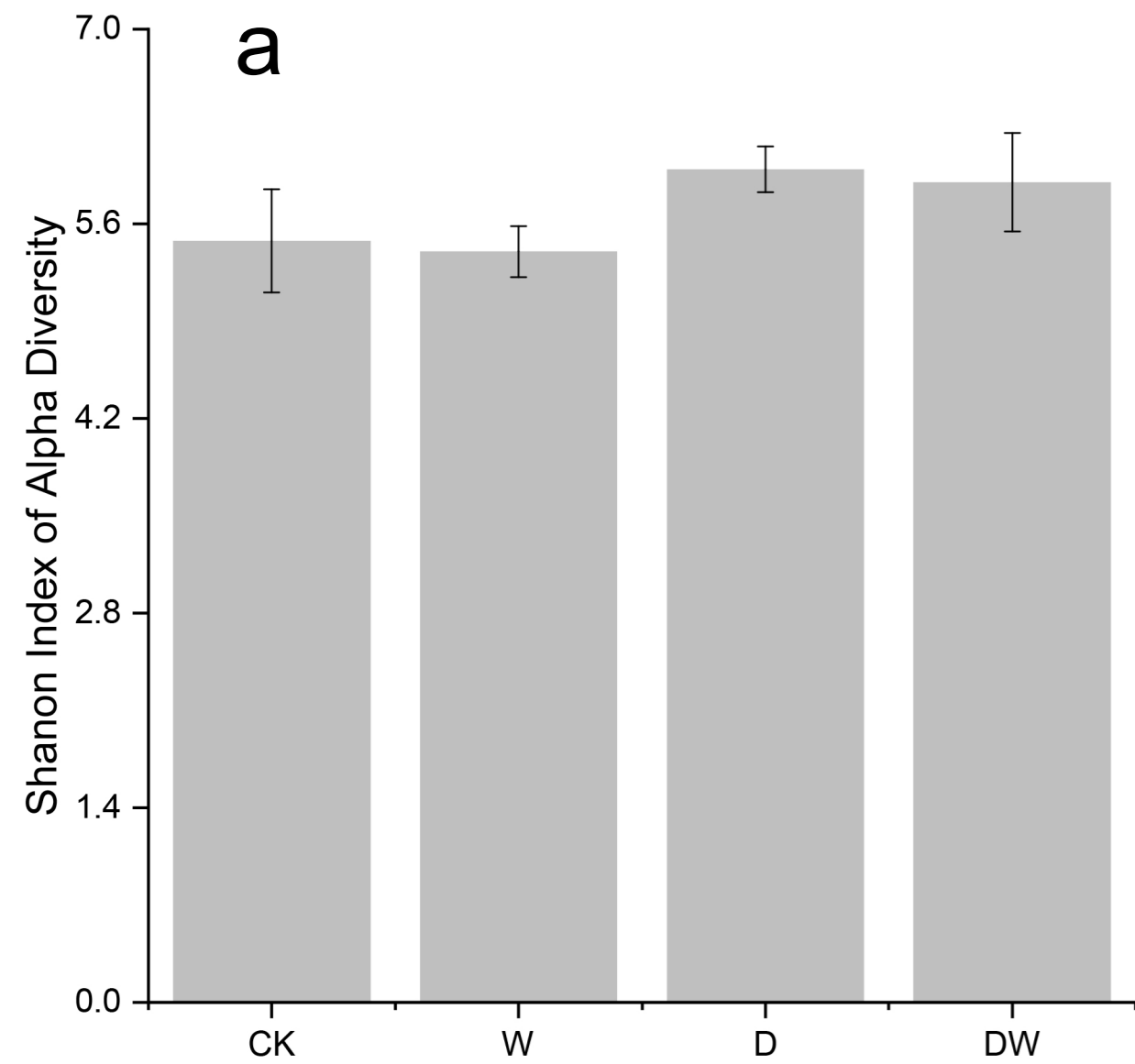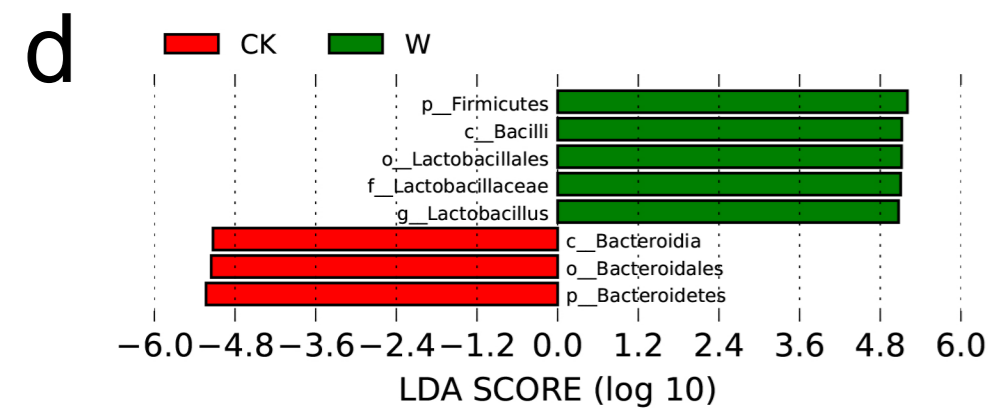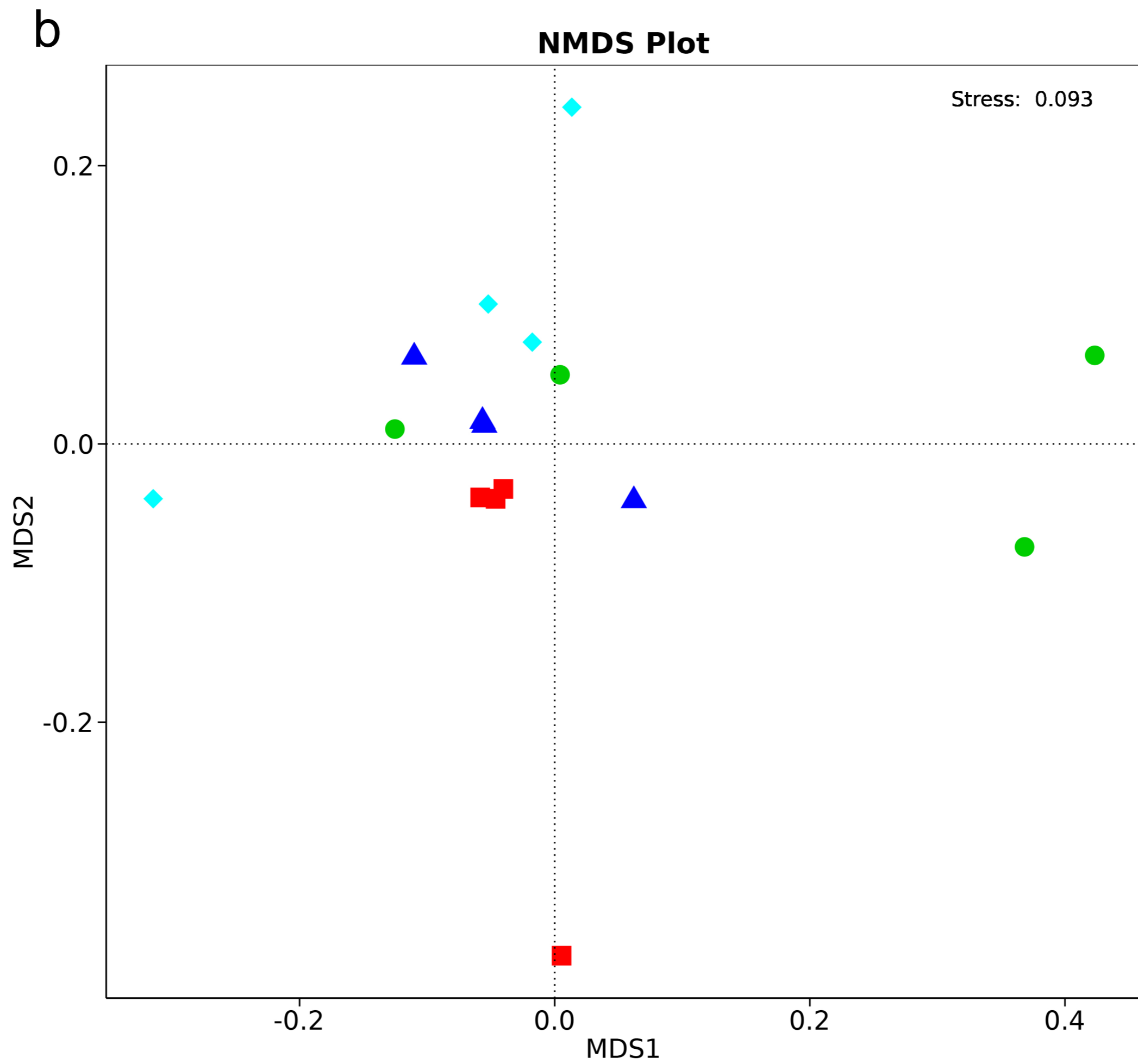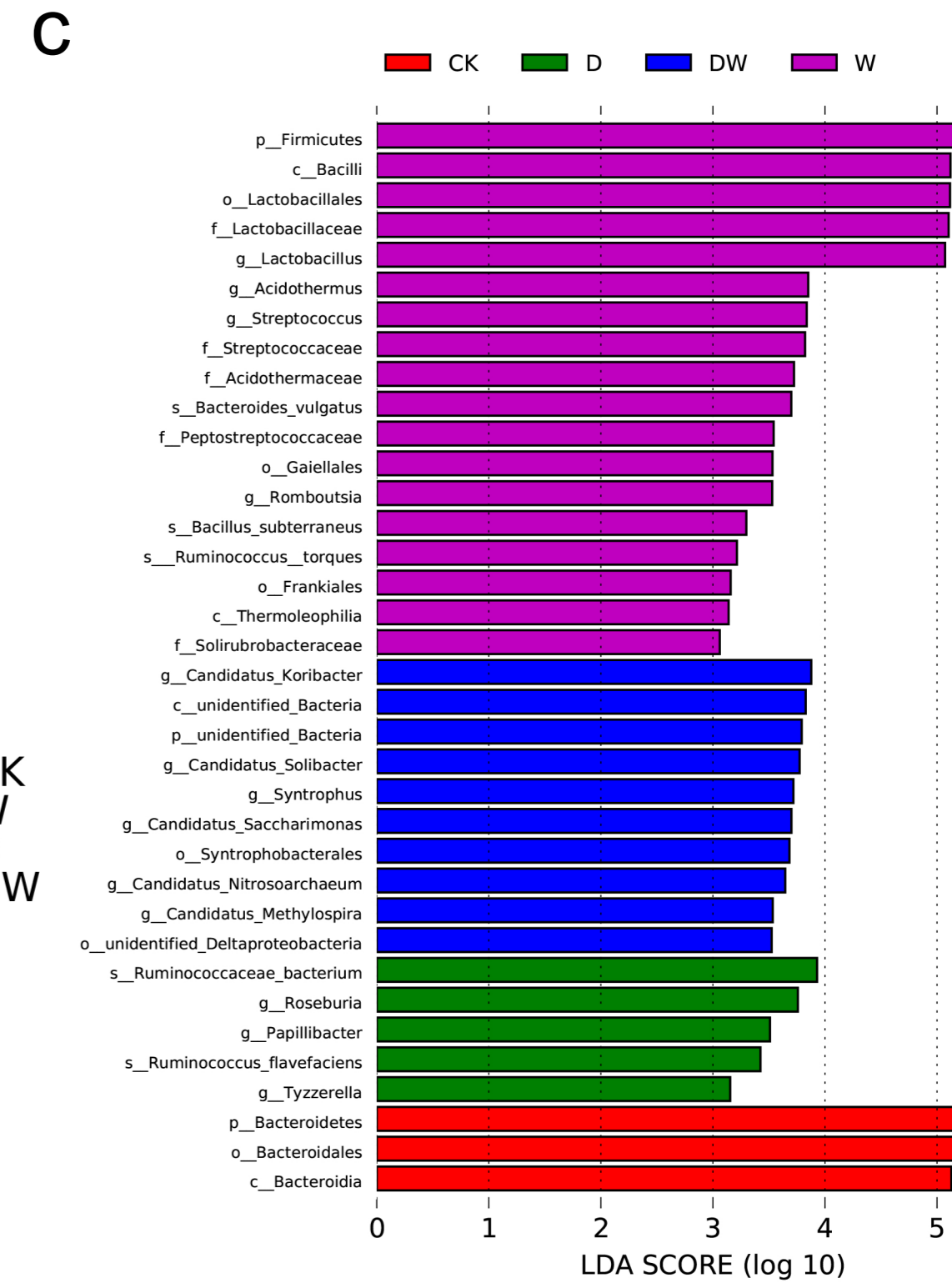

Supplement: Supplementary file 1 [file foods-10-00835-s001.zip › supplementary files/Supplementary File 6ú║Fig. S5.pdf]

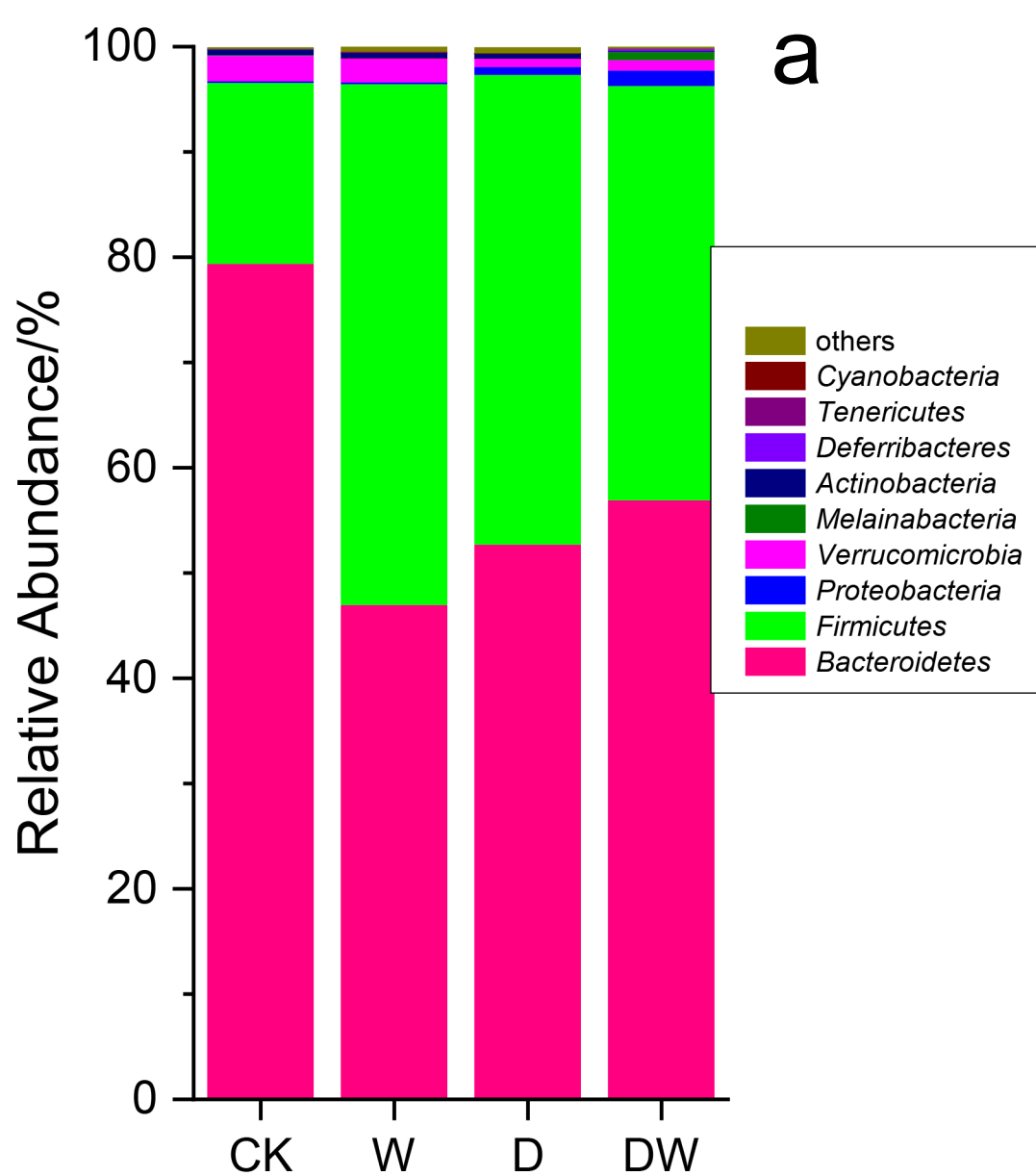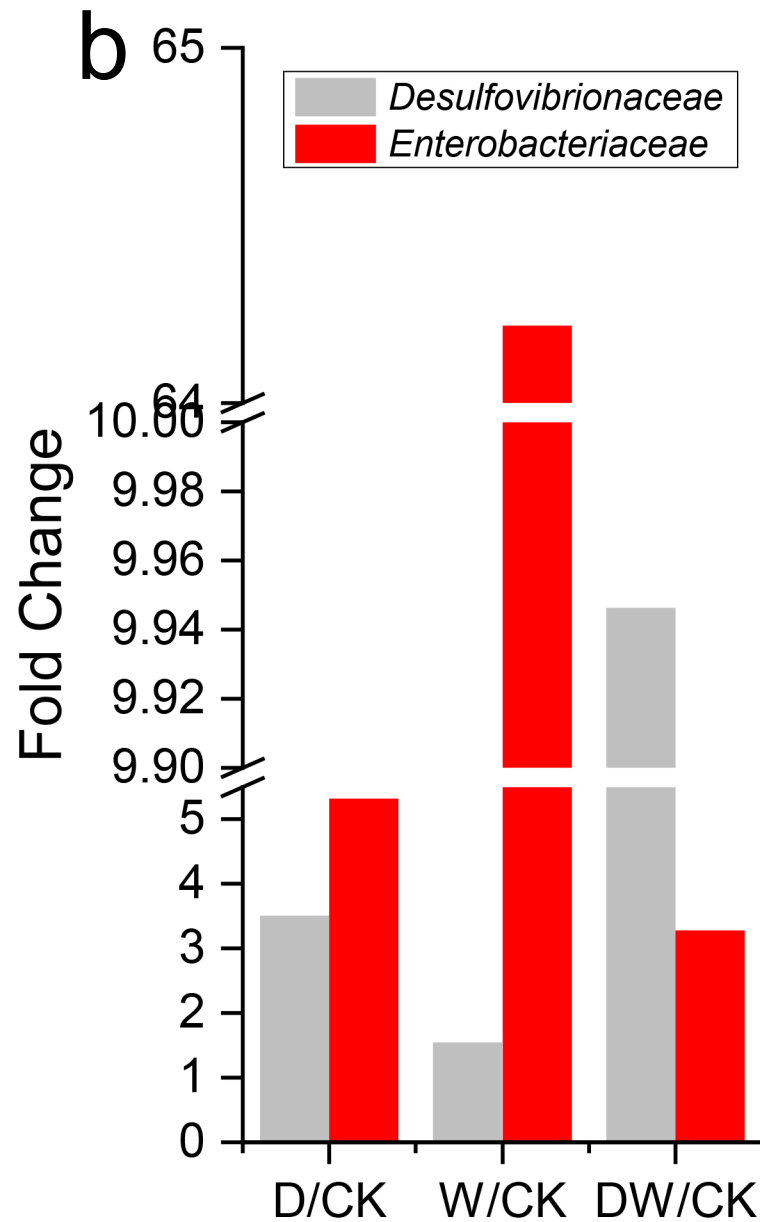

Supplement: Supplementary file 1 [file foods-10-00835-s001.zip › supplementary files/Supplementary File 7ú║Fig. S6.pdf]

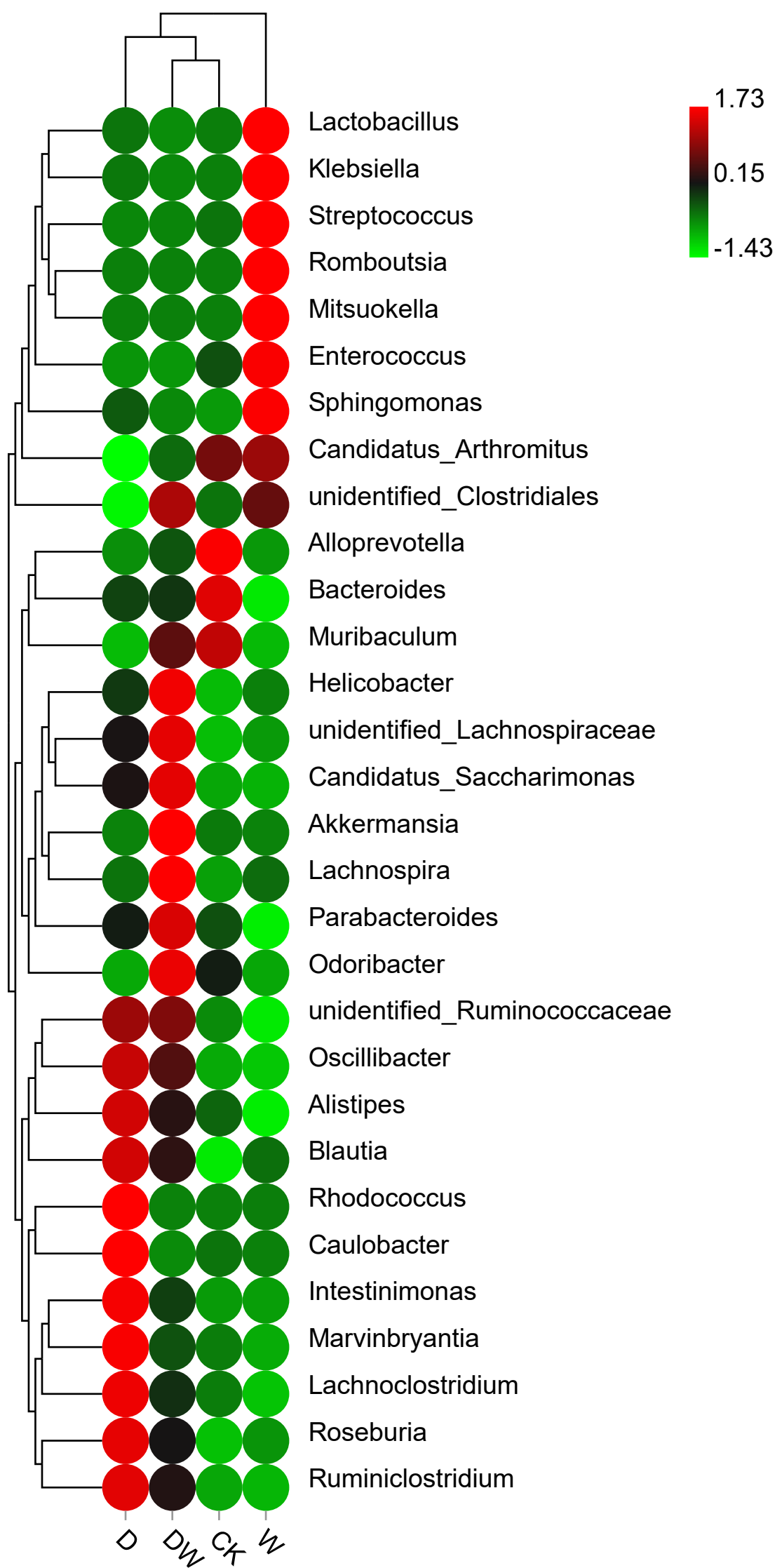

Supplement: Supplementary file 1 [file foods-10-00835-s001.zip › supplementary files/Supplementary File 8ú║Fig. S7.pdf]

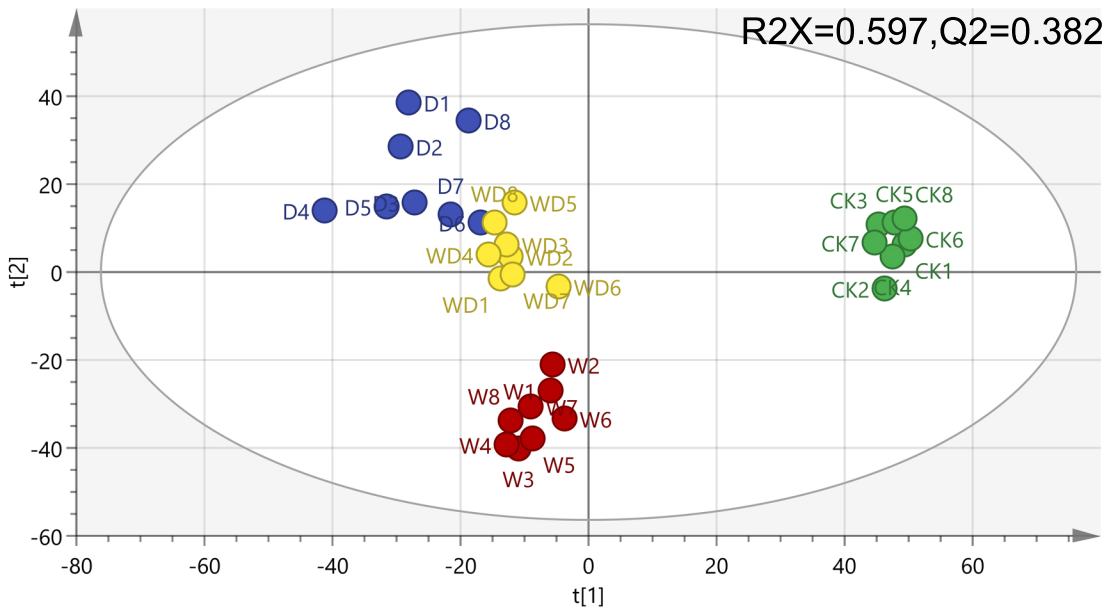

Supplement: Supplementary file 1 [file foods-10-00835-s001.zip › supplementary files/Supplementary File 9ú║Fig. S8.pdf]
